# Developmentally Inspired, Mechanical–Metabolic Dual Gradient Osteochondral Constructs Bridging Regeneration and Therapeutic Screening

**DOI:** 10.1002/advs.202516602

**Published:** 2026-03-03

**Authors:** Yurim Choi, Wonjun Jang, Raehui Kang, Ulziituya Batjargal, Jihyeon Song, Soojin Park, MinSeok Kim, Hyobum Cho, Junhyung Kim, Yu Shrike Zhang, Han‐Jun Kim, Junmin Lee

**Affiliations:** ^1^ Department of Materials Science and Engineering Pohang University of Science and Technology (POSTECH) Pohang Republic of Korea; ^2^ College of Pharmacy Korea University Sejong Republic of Korea; ^3^ Interdisciplinary Major Program in Innovative Pharmaceutical Sciences Korea University Sejong Republic of Korea; ^4^ Division of Interdisciplinary Bioscience & Bioengineering Pohang University of Science and Technology (POSTECH) Pohang Republic of Korea; ^5^ College of Veterinary Medicine and Institute of Veterinary Science Kangwon National University Chuncheon Republic of Korea; ^6^ Division of Engineering in Medicine, Department of Medicine Brigham and Women's Hospital, Harvard Medical School Cambridge USA; ^7^ Department of Plastic and Reconstructive Surgery, College of Medicine Korea University Seoul Republic of Korea

**Keywords:** 3D bioprinting, drug screening, dual gradients, mechanotransduction, mesenchymal stem cells, osteoarthritis, osteochondral interface

## Abstract

The osteochondral interface is a finely tuned junction between cartilage and bone, coordinated by gradients in mechanics and metabolism. Recreating this complexity in vitro has remained elusive. Here, we present a developmentally inspired, dual‐gradient 3D‐printed construct that unites native‐like stiffness and metabolic microenvironments to drive spatially resolved regeneration and model osteoarthritis in a phase‐specific manner. Human bone marrow‐derived mesenchymal stem cell spheroids are placed in soft, hypoxic niches to promote chondrogenesis, and in stiff, vascular‐rich regions to induce osteogenesis—preserving cartilage–bone crosstalk within one platform. This integration of gradients amplifies extracellular matrix formation beyond single‐cue designs through the synergistic effect of porosity and stiffness. Furthermore, its anisotropic architecture maintains cartilage–bone crosstalk and allows drug response assessment, highlighting its potential as a physiologically relevant platform for osteoarthritis modeling and therapy screening. By bridging functional regeneration with preclinical drug screening, this model offers a physiologically relevant translational platform for advancing both osteochondral repair and disease research.

## Graphical abstract

A developmentally inspired osteochondral construct integrates mechanical and metabolic dual gradients within a 3D‐printed platform. Human MSC spheroids are spatially directed to form cartilage–bone interfaces, enabling evaluation of regeneration without biochemical reagents and assessment of drug responses. This physiologically relevant, phase‐specific osteoarthritis model offers a translational tool for regenerative research and preclinical screening.

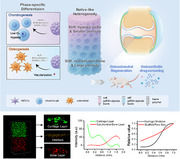

## 1 Introduction

The osteochondral (OC) interface is a structurally and functionally heterogeneous region that bridges the rigid subchondral bone to the soft articular cartilage [1]. As a transitional zone between mechanically and biologically distinct tissues, it exhibits continuous gradients in stiffness, cellular composition, oxygen concentration, nutrient diffusion, and vascularization [2]. This intricately organized interface facilitates mechanical load transfer and contributes to joint homeostasis. However, it is highly susceptible to degeneration under repetitive stress, particularly with aging, obesity, or trauma [3]. Such degeneration can lead to osteoarthritis (OA), a chronic and debilitating condition that affects over 500 million people worldwide [4]. Conventional surgical treatments, including microfracture and autologous grafting, often fail to regenerate durable hyaline cartilage and suffer from poor integration with native tissue [5, 6]. As a result, many patients continue to experience pain and limited joint function, and some ultimately require additional surgical intervention. These limitations have prompted a shift toward tissue engineering strategies aimed at recapitulating physiological structure and functions, rather than simply filling defects [2, 3].

Owing to their capacity for lineage‐specific differentiation, human bone marrow‐derived mesenchymal stem cells (hMSCs) are considered one of the most promising cell sources for osteochondral tissue engineering [7]. Conventional strategies have primarily relied on the delivery of exogenous growth factors (e.g., TGF‐β, BMP‐2) or small molecules to direct hMSCs fate [8, 9, 10]. While these biochemical strategies can induce transient lineage commitment, they are fundamentally limited by multiple critical drawbacks. These cues typically exhibit short half‐lives and burst‐release kinetics in vivo [11, 12], often leading to supraphysiological concentrations that can trigger unintended differentiation or off‐target effects [13]. Also, their instability often necessitates complex delivery systems and repeated dosing, further increasing production costs and regulatory hurdles [14]. Most importantly, these conventional strategies primarily focus on cell‐intrinsic programming while largely overlooking the essential role of the native microenvironment's heterogeneity— spatially organized mechanical and metabolic gradients—that are vital for guiding functional tissue regeneration. These challenges highlight the need for biomaterial systems that can recapitulate physiologically relevant niches and support spatially directed, long‐term regeneration.

During embryonic development, formation of the native osteochondral interface is orchestrated by a mechano–metabolic axis in which mechanical forces and metabolic gradients work in concert to regulate progenitor cell fate [15, 16]. Stiffness and elasticity cues are transduced through YAP/TAZ [17] and integrin–FAK signaling [18] to control cytoskeletal organization, ECM assembly, and spatial differentiation—rigid regions driving osteogenesis and compliant regions promoting mesenchymal condensation and chondrogenesis [19, 20, 21, 22]. In parallel, oxygen and nutrient gradients further refine these spatial patterns. Hypoxic conditions within the avascular cartilage zone stabilize HIF‐1α, sustaining SOX9‐driven chondrogenesis [23], while normoxic, well‐vascularized bone regions enhance RUNX2‐mediated osteogenesis and mineralization [24]. Mechanical and metabolic cues act synergistically, and these dynamic gradients create the spatial organization necessary for seamless cartilage–bone integration, providing a critical blueprint for the design of functional osteochondral constructs.

**FIGURE 1:**
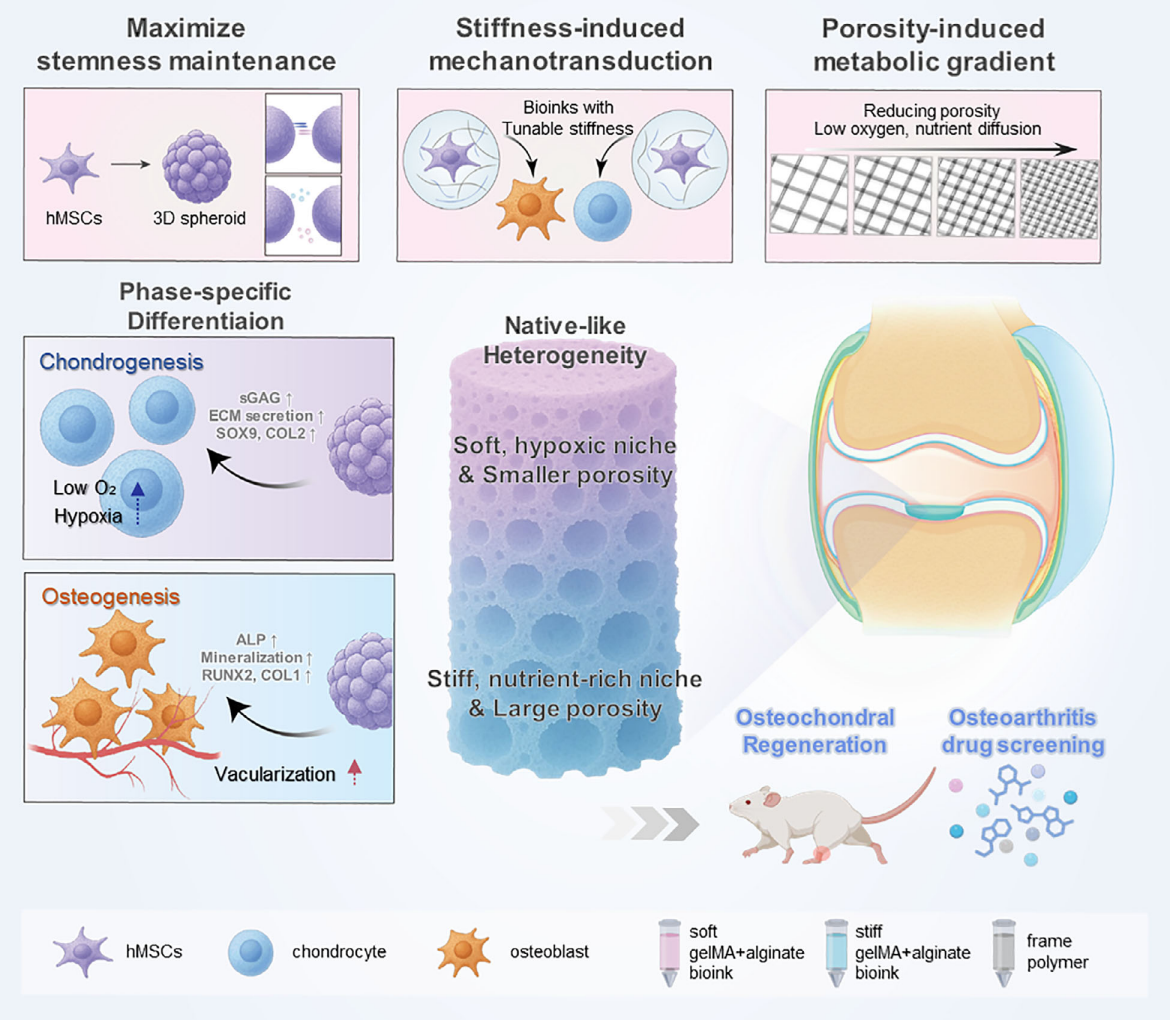
Graphical overview of osteochondral tissue engineering strategy integrating biological, mechanical, and metabolic cues.

Inspired by developmental principles, we developed a biomaterial‐based strategy that replicates key biomechanical and metabolic gradients in a single construct. (Figure 1) To improve lineage fidelity, hMSCs were embedded as 3D spheroids, mimicking the initial condensation phase of development [21, 25]. Using a multi‐material 3D printing platform, we engineered a construct with a spatially tunable stiffness gradient—achieved by combining gelatin methacryloyl (GelMA) with stress‐relaxing alginate—and a metabolic gradient established through a porous polycaprolactone (PCL) framework featuring pore sizes ranging from 300 to 900 µm. Notably, the integration of mechanical and metabolic gradients leads to synergistic effect, enhancing matrix deposition and functional tissue formation beyond that of single‐cue systems. Unlike conventional hydrogel‐only printing approaches, which often lack mechanical robustness, our platform provides tissue‐mimetic mechanical properties that help prevent delamination and reduce inflammation caused by interfacial mismatch under dynamic stresses in vivo [26, 27]. Furthermore, its mature anisotropic structure preserves cartilage–bone crosstalk and enables the assessment of drug responses, demonstrating its utility as a pathophysiologically relevant platform for osteoarthritis modeling and therapeutic screening. Altogether, this work introduces a developmentally inspired strategy for osteochondral regeneration and disease modeling, offering a next‐generation system that integrates mechanical and metabolic cues to recapitulate native tissue function.

## 2 Results and Discussion

### 2.1 Optimization of hMSC Spheroid Formation for Stemness Maintenance

**FIGURE 2:**
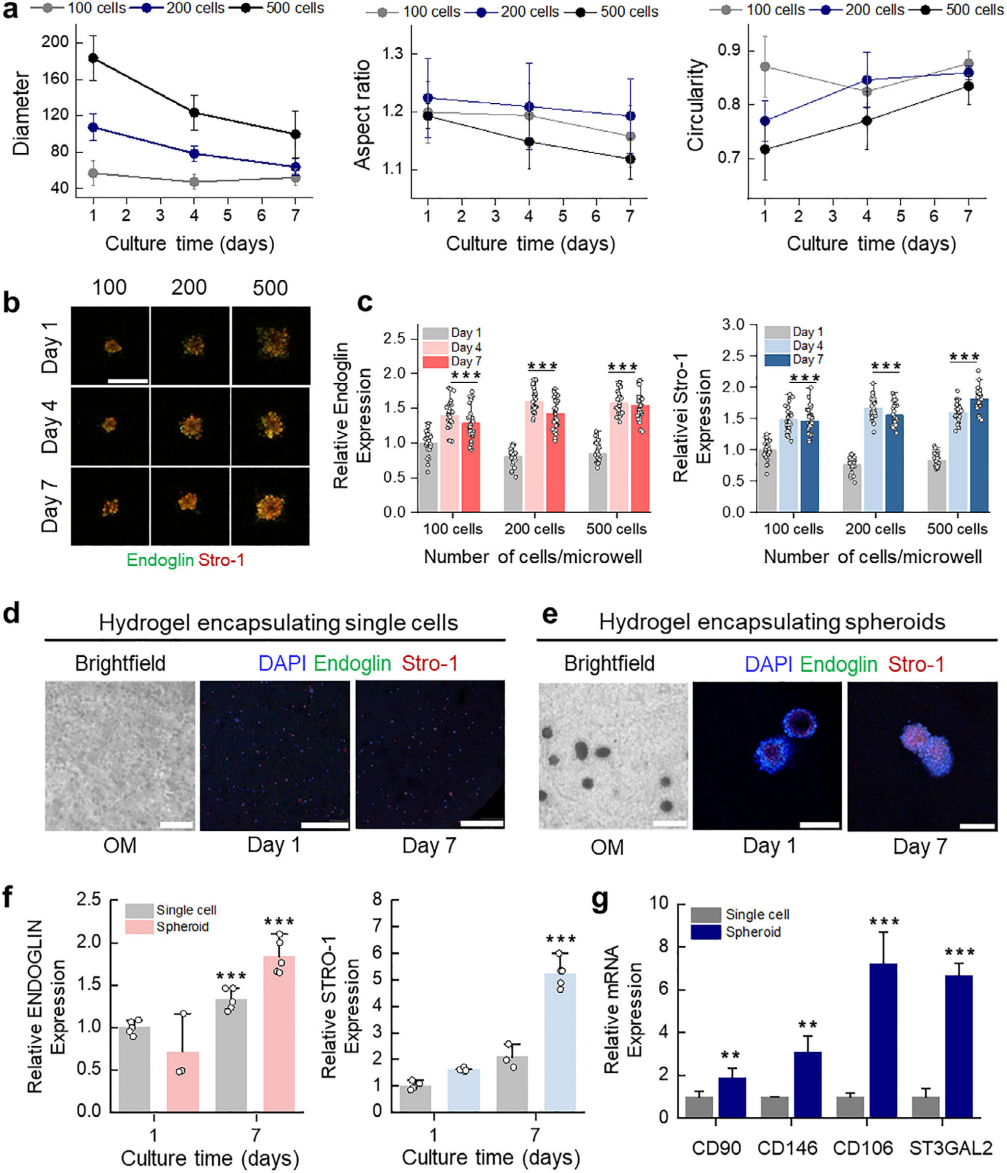
Morphological characterization and stemness maintenance of hMSC spheroids. a) Quantitative analysis of spheroid diameter aspect ratio, and circularity over 7 days of culture at 100, 200, and 500 cells/microwell (n = 6). b) Representative immunofluorescence images for Stro‐1 and Endoglin. Scale bar: 200 µm. c) Relative Endoglin and Stro‐1 expression levels, normalized to the 100‐cell group at day 1 (n = 30). Statistical significance was calculated relative to day 1. d) Representative bright‐field showing encapsulated cells and immunofluorescence images showing Endoglin (red) and Stro‐1 (green) expression in single‐cell and spheroid cultures; nuclei were counterstained with DAPI (blue). Scale bars: 100 µm for brightfield, 200 µm for single cell confocal image and 50 µm for spheroid confocal image. f) Relative expressions of Endoglin and Stro‐1 in single‐cell vs spheroid culture. (n ≥ 3). Statistical significance was calculated relative to day 1. g) qRT‐PCR analysis of stemness‐ and clinically relevant markers (n = 3). Statistical significance was calculated relative to single cell expression. Data are presented as mean ± SD. Statistical significance was determined by one‐way ANOVA with Tukey's post–hoc test; ^*^ *p* < 0.05, ^#^ *p* < 0.01, ^**^ *p* < 0.005, ^***^ *p* < 0.0005.

hMSCs are a fundamental cell source in this study due to their multipotent differentiation capacity. However, when administered as a single‐cell suspension, hMSCs rapidly undergo loss after implantation because of anoikis and inflammatory responses, and they often lose stemness or differentiation potential before transplantation, posing a significant clinical challenge [28, 29]. To address these issues, we generated spheroids that mimic the condensation phase of development, thereby enhancing cell–cell adhesion and preserving stemness [30, 31]. We employed a microwell‐based culture system to generate uniform, size‐controllable hMSC spheroids at different seeding densities (100, 200, and 500 cells/microwell). At 1000 cells per well, excessive cell proliferation led to overflow, disrupting spheroid formation (Figure S1). Morphological parameters, including diameter, circularity, and aspect ratio, were evaluated from fluorescent‐labeled spheroids on Days 1, 4, and 7 (Figure 2a; Figure S2) For the 100 cells/microwell condition, the diameter, aspect ratio, and circularity did not change significantly over time. In contrast, spheroids formed from 200 and 500 cells/microwell contracted progressively until Day 4 and then plateaued, with a greater reduction in the 500‐cell condition. Specifically, diameters decreased from 107 to 63 µm (1.7‐fold) for 200 cells, and from 183 to 99 µm (1.9‐fold) for 500 cells This contraction indicates increased cell–cell interactions over time, which are critical for stemness maintenance and cell survival by activating key pathways (Wnt, Notch, TGF‐β) similar to the natural stem cell niche [32, 33]. In terms of circularity, there were no significant differences between the 200‐ and 500‐cell conditions; however, the aspect ratio was closer to 1 in the 500‐cell group at Day 7 (1.118 ± 0.03 vs 1.19 ± 0.06), suggesting higher uniformity and symmetry. This uniformity and symmetry are important factors for ensuring consistent nutrient and oxygen diffusion throughout the spheroid, helping to maintain spheroid integrity. Stemness marker analysis further supported these findings (Figure 2b). Stro‐1, a primitive hMSC marker associated with self‐renewal and multilineage differentiation, and Endoglin, linked to multipotency, both increased in expression over time in all groups, plateauing between Days 4 and 7 (Figure 2c). Notably, the 100‐cell spheroids exhibited the smallest magnitude of increase, whereas both the 200‐ and 500‐cell spheroid conditions showed significantly greater upregulation compared to the 100‐cell condition. No statistically significant difference was observed between the 200‐ and 500‐cell groups. (Figure S3). Therefore, considering both stemness markers and morphological aspects, we optimized the condition with 500 cells per microwell on Day 4, as it demonstrated the highest and most stable stemness.

To assess whether enhanced stemness was due to cell–cell interactions rather than the 3D environment alone, we compared single cells encapsulated in hydrogels (Figure 2d) with spheroids encapsulated in hydrogels (Figure 2e), both with the same cell density. To minimize the risk of contamination, we employed thoroughly sterilized PDMS molds, which enabled reproducible and uniform cell encapsulation (Figure S4). As culture time increased, the single‐cell group showed a slight increase in Endoglin (1.3‐fold) and Stro‐1 (1.61‐fold) expression at day 7 compared to day 1. In contrast, spheroids exhibited 2.14‐ fold increases in Endoglin, and 2.66‐fold increases in Stro‐1 expression. (Figure 2f) This highlights that cell–cell interaction within spheroids plays a more significant role in enhancing stem cell functionality than merely using a 3D environment. Finally, gene expression analysis by qPCR revealed higher levels of stemness‐ and clinically relevant markers in spheroids compared to single cells (Figure 2g). CD90 and ST3GAL2 are all upregulated in spheroid groups. These genes are associated with stemness, cell adhesion, and interactions with immune cells, which contribute to the stability, engraftment, and immune modulation of transplanted cells [34, 35]. Notably, CD146, a vascular marker linked to tissue homing, and CD106, a key adhesion molecule for hMSC–host interaction, were markedly elevated in spheroids [36, 37]. These results underscore the clinical relevance of hMSC spheroids, not only for their differentiation capacity but also for their potential to modulate host responses and improve engraftment efficiency compared to single cell preparations.

### 2.2 Mechanical and Rheological Performance of GelMA–Alginate Bioinks/PCL Scaffolds

hMSCs sense and respond to the stiffness of their microenvironment, directing lineage‐specific differentiation through mechanotransduction. Softer substrates promote chondrogenic gene expression by reducing cytoskeletal tension, whereas stiffer substrates enhance actomyosin contractility, YAP/TAZ nuclear localization for osteogenesis [38]. Based on these principles, we optimized the stiffness of the encapsulating bioink to provide lineage‐specific cues. Gelatin methacryloyl (GelMA) was selected as a primary component due to its inherent bioactivity, presence of RGD motifs that promote cell adhesion, and tunable mechanical properties via degree of methacrylation and UV crosslinking (Figure S5) [39]. Alginate was incorporated as a secondary component to improve structural integrity, modulate stiffness and stress‐relaxation through ionic crosslinking. This dual‐network design allows fine‐tuning of viscoelasticity to match the mechanical cues required for targeted hMSC differentiation [40].

**FIGURE 3:**
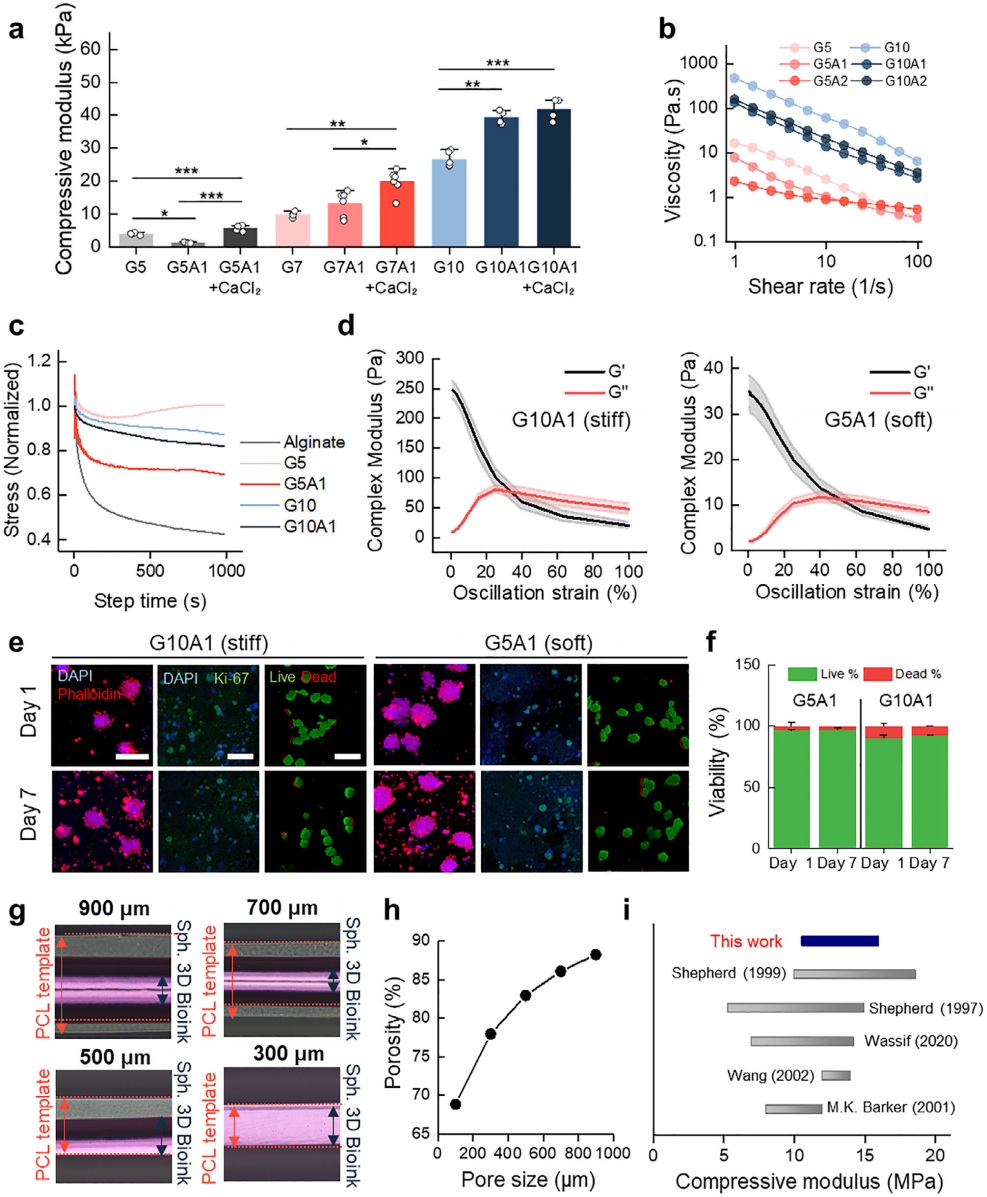
Mechanical, rheological, and structural characterization of GelMA–alginate hybrid bioinks and PCL template structures. a) Compressive modulus of GelMA and alginate‐containing hydrogels (n ≥ 3). b) Shear‐thinning behavior of bioinks at varying shear rates (n = 3). c) Stress–relaxation behavior of GelMA‐only and GelMA–alginate hybrid hydrogels (n = 3) d) Strain‐dependent rheological behavior showing storage modulus (*G′*) and loss modulus (*G″*) of stiff (G10A1) and soft (G5A1) hydrogels. e) Representative confocal images of F‐actin (phalloidin, magenta), nuclei (DAPI, blue), Ki‐67 (green) staining, and Live/Dead assay results. Scale bars: 200 µm for F‐actin, 100 µm for Ki‐67 and Live/Dead assay. f) Quantitative viability analysis of encapsulated hMSCs spheroids (n = 3). g) Representative top‐view images of hybrid printed construct with varying pore sizes (900, 700, 500, and 300 µm). g) Porosity of 3D‐printed scaffolds as a function of pore size. i) Comparison of the compressive modulus of PCL templates in this work with native tissue modulus reported in previous studies. Data are presented as mean ± SD.

Icreasing GelMA concentration elevated the compressive modulus, and alginate addition further reinforced stiffness via a secondary ionic network. (Figure 3a; Figure S6). Previous studies have demonstrated that hydrogels with a modulus of approximately ∼10 kPa promote chondrogenesis, whereas stiffer matrices of ∼30 kPa favor osteogenic differentiation [41, 42, 43, 44, 45]. Based on these findings, we designated the 5% GelMA with 1% alginate (G5A1) formulation as the chondrogenic ink and the 10% GelMA with 1% alginate (G10A1) formulation as the osteogenic ink. The addition of alginate to GelMA diluted the GelMA polymer network, reducing chain entanglement and thereby lowering viscosity prior to Ca^2^^+^ crosslinking. Because alginate has a relatively low intrinsic viscosity before ionic gelation, its inclusion slightly decreased the overall viscosity while maintaining the shear‐thinning behavior of the bioink. (Figure 3b). The sol–gel transition temperature of GelMA‐based bioinks increased with alginate content, as determined by temperature sweep rheometry (Figure S7). This enhanced thermal stability also improves the performance of the bioink during the printing process by maintaining its structural integrity over a wider temperature range. Based on the measured sol–gel transition temperature, the printing head temperature was set to 20°C to ensure optimal extrusion while preserving the bioink's structural stability during deposition. Incorporating ionically crosslinked alginate markedly improved stress relaxation (Figure 3c). Increased stress relaxation is advantageous for cell‐laden bioinks, as it reduces sustained mechanical stress on encapsulated cells, facilitates cell‐mediated matrix remodeling, and more closely mimics the viscoelastic properties of native tissues [46]. Strain sweep tests confirmed that *G′* exceeded *G″* up to 10% strain, maintaining structural integrity under physiologically relevant deformation (Figure 3d) [47]. The frequency sweep showed that *G′* exceeded *G″* at all tested frequencies for both bioinks (Figure S8). This indicates a predominantly elastic, solid‐like behavior with enhanced structural rigidity under dynamic loading. To further evaluate the temporal stability of the bioinks, an in vitro accelerated degradation assay was conducted. (Figure S9). The hydrogel bioinks degraded substantially faster than the PCL scaffold, with softer formulations exhibiting more rapid mass loss, indicating an early creation of space for cell infiltration while maintaining structural support from the slowly degrading PCL framework.

Biocompatibility of the bioinks was evaluated through cell spreading, proliferation, and viability over 7 days (Figure 3e). For spheroid‐encapsulating bioinks, a cell density of 6 × 10^6^ cells/mL (corresponding to 1200 spheroids/mL) was selected as an optimized condition that allows sufficient cell loading while maintaining the printability and structural integrity of the hydrogel bioink. At this density, the bioink retained suitable rheological properties for extrusion‐based printing and supported adequate cell density required for early tissue development and integration with host tissue. After printing process, hMSC spheroids maintained their compact morphology over time, reflecting strong cell–cell interactions (Figure S10a). A slight increase in spheroid area was observed in the soft hydrogel, whereas no notable change occurred in the stiff hydrogel, suggesting that the softer environment may allow limited outward cell migration while the stiffer matrix constrains expansion. Proliferation analysis revealed that more than 80% of cells were Ki‐67–positive in both bioinks, with no decrease over time (Figure S10b), indicating sustained proliferative capacity. Also, both formulations maintained over 90% cell viability throughout the 7‐day culture period (Figure 3f). Collectively, these results demonstrate that bioinks in this study provide a highly cytocompatible environment capable of supporting long‐term cell survival and proliferation while preserving the spheroid architecture.

While GelMA‐ and alginate‐based hydrogels are favorable for cell encapsulation, their mechanical strength is substantially lower than that of native tissues, and such softness limits their suitability for precisely designing porosity gradients. To overcome this limitation, we employed a hybrid printing strategy in which cell‐laden hydrogel was co‐printed with polycaprolactone (PCL), a material with high mechanical strength and excellent printing fidelity. For controlled oxygen and nutrient gradients, PCL was printed with gradually decreasing pore sizes of 900, 700, 500, and 300 µm, with bioink layers deposited between the PCL layers (Figure 3g; Figure S11). As pore size increased, scaffold porosity also increased (Figure 3h; Figure S12), which is expected to generate metabolic gradients within the construct. Cross‐sectional, top, and bottom view optical microscopy images confirmed the structural integrity and spatially graded pore architecture of the printed osteochondral scaffold (Figure S13).

During fabrication of the PCL–bioink composite construct, the high processing temperature required for PCL extrusion may pose a risk of thermal damage to the hydrogel bioink and encapsulated cells. To mitigate this risk, the PCL framework and the hydrogel‐based bioink were printed sequentially using separate printheads with different nozzle diameters (400 µm for PCL and 250 µm for the bioink). This configuration resulted in a controlled difference in layer height, ensuring that the PCL nozzle did not come into direct contact with the previously deposited bioink layers during subsequent PCL printing. To assess the potential impact of hybrid printing on cell survival, viability was quantified under conditions in which the thermoplastic extrusion head was positioned in closest proximity to the encapsulated cells. The smallest pore size (300 µm) was selected as the representative condition for evaluation. As shown in Figure S14, hybrid‐printed constructs exhibited greater than 80% cell viability, which was comparable to that of hydrogel‐only printed counterparts. The full‐thickness construct achieved a compressive modulus within the native osteochondral range (Figure 3i), minimizing mechanical mismatch and reducing risks of delamination or inflammatory responses, thereby enhancing integration and long‐term stability [48].

### 2.3 Synergistic Effects of Stiffness and Porosity on Spatially Programmed Differentiation

**FIGURE 4:**
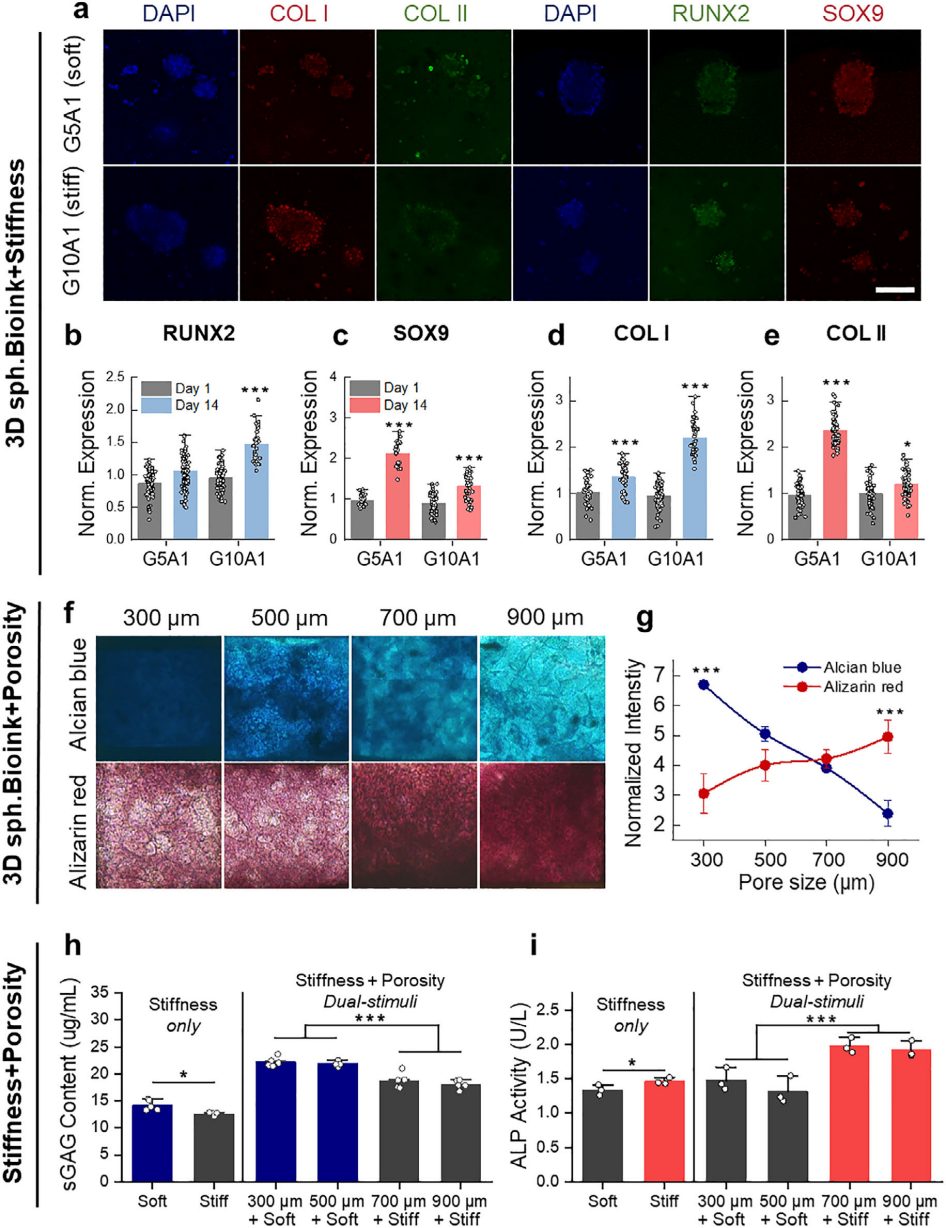
Effect of bioink stiffness and porosity on chondrogenic and osteogenic differentiation of hMSC spheroids. a) Representative immunofluorescence images of chondrogenic (COL I, COL II) and osteogenic (RUNX2, SOX9) markers in spheroids cultured in stiff and soft bioinks for 14 days; nuclei stained with DAPI (blue). Scale bars: 100 µm. b–e) Quantification of normalized expression of RUNX2 (b), SOX9 (c), COL I (d), and COL II (e) at days 1 and 14 (n ≥ 20). Statistical significance was calculated relative to Day 1. f) Representative images of alcian blue and alizarin red S staining in spheroids cultured within bioinks printed in PCL scaffolds of different pore sizes (300, 500, 700, and 900 µm). g) Quantitative analysis of normalized staining intensity for glycosaminoglycan (Alcian blue) and calcium deposition (Alizarin Red) (n = 3). Statistical significance was calculated relative to biggest porosity for alcian blue and smallest porosity for alizarin red. h) Quantification of sulfated glycosaminoglycan (sGAG) content in spheroids cultured in bioinks with different stiffness and scaffold porosities (n = 5). i) Quantification of alkaline phosphatase (ALP) activity (U/L) in the same groups (n = 3). Data are presented as mean ± SD. Statistical analysis was performed using one‐way ANOVA with Tukey's post–hoc test; ^*^ *p* < 0.05, ^#^ *p* < 0.01, ^**^ *p* < 0.005, ^***^ *p* < 0.0005.

To test our hypothesis that stem cell fate can be directed toward tissue‐specific lineages in situ without the need for exogenous biochemical reagents, we focused on engineering the local microenvironment to recapitulate biophysical and metabolic features of native tissues. Specifically, we aimed to investigate whether variations in microenvironmental stiffness and porosity—either individually or in combination—could guide hMSCs behavior and lineage commitment. As an initial step, we examined the effect of bioink stiffness on hMSCs differentiation. To determine whether the stiffness differences in our hydrogel system are translated into intracellular signaling, we first examined YAP localization in hMSCs cultured on hydrogels with different mechanical properties. YAP was predominantly localized in the cytoplasm on the soft G5A1 hydrogel, whereas clear nuclear localization was observed on the stiff G10A1 hydrogel. Quantitative analysis confirmed a higher nuclear‐to‐cytoplasmic YAP ratio on G10A1 (2.65 ± 0.43) compared to G5A1 (1.59 ± 0.41), indicating enhanced mechanotransduction in response to increased matrix stiffness (Figure S15). Based on this mechanistic difference, we next evaluated how hydrogel stiffness influences lineage commitment of hMSC spheroids cultured under basal conditions using immunofluorescence staining (Figure 4a). We analyzed the expression of key osteogenic and chondrogenic markers at both early and late stages. RUNX2 and SOX9, master transcription factors associated with osteogenic and chondrogenic commitment respectively, were used to assess early‐stage specifications. Their expression patterns varied depending on matrix stiffness: RUNX2 expression was significantly elevated in stiff hydrogels (Figure 4b), whereas SOX9 was more highly expressed in soft substrates (Figure 4c). To assess late‐stage matrix formation, we examined the expression of COL1 and COL2, structural components of bone and cartilage ECM, respectively. Consistent with early marker expression, COL1 levels were higher in stiff conditions (Figure 4d), while COL2 expression was upregulated in soft hydrogels (Figure 4e), indicating stiffness‐dependent matrix deposition aligned with lineage‐specific differentiation. Additionally, RUNX2, a marker associated with hypertrophic cartilage, did not increase during the 14‐day culture period in soft hydrogels [49]. This indicates that encapsulated spheroids differentiated toward hyaline cartilage rather than undergoing hypertrophy, highlighting the role of soft hydrogels in maintaining cartilage phenotype. The coordinated upregulation of transcription factors and matrix proteins underscores the importance of mechanical cues in driving robust, developmentally relevant tissue specification.

We next investigated how scaffold porosity modulates functional extracellular matrix (ECM) production in hydrogels with controlled stiffness. Alizarin Red S staining was used to visualize calcium deposits indicative of osteogenesis, while Alcian Blue staining detected sulfated glycosaminoglycans (sGAGs), a hallmark of cartilage ECM. In G5A1 hydrogels, a decrease in porosity led to a increased deposition of sulfated glycosaminoglycans (sGAGs) within the matrix (Figure 4f). This suggests that confined, low‐porosity environments promote chondrogenesis by mimicking the hypoxic, nutrient‐limited milieu of native cartilage. In contrast, in G10A1 hydrogels, higher porosity correlated with stronger Alizarin Red S staining, reflecting enhanced calcium deposition associated with osteogenic differentiation. This suggests that in mechanically rigid environments, increased porosity facilitates improved nutrient and oxygen transport, which in turn supports osteoblast activity and mineralization Quantitative image analysis confirmed graded increases in lineage‐specific ECM production with porosity changes, even when stiffness was held constant (Figure 4g).

Finally, we evaluated the combined effects of stiffness and porosity gradients on lineage‐specific differentiation. Specifically, we compared stiffness‐only conditions (without porosity) with constructs incorporating both stiffness and graded porosity, evaluating sGAG content and ALP activity as indicators of chondrogenic and osteogenic responses, respectively (Figure 4h,i). As previously observed, stiffness alone promoted lineage‐specific matrix production: soft hydrogels supported greater sGAG accumulation, while stiff hydrogels induced higher ALP activity. When porosity gradients were incorporated, smaller pores (300–500 µm) in soft hydrogels significantly enhanced sGAG content (Figure 4h), while larger pores (700–900 µm) in stiff hydrogels elevated ALP activity (Figure 4i). These findings underscore that mechanical and metabolic gradients act synergistically to achieve spatially resolved differentiation within a single construct, recapitulating native tissue architecture and function. Based on these results, the upper cartilage layer was assigned to soft hydrogels with 300–500 µm pores, and the lower subchondral layer to stiff hydrogels with 700–900 µm pores.

### 2.4 Angiogenic and Metabolic Gradients for Functional Osteochondral Regeneration

**FIGURE 5:**
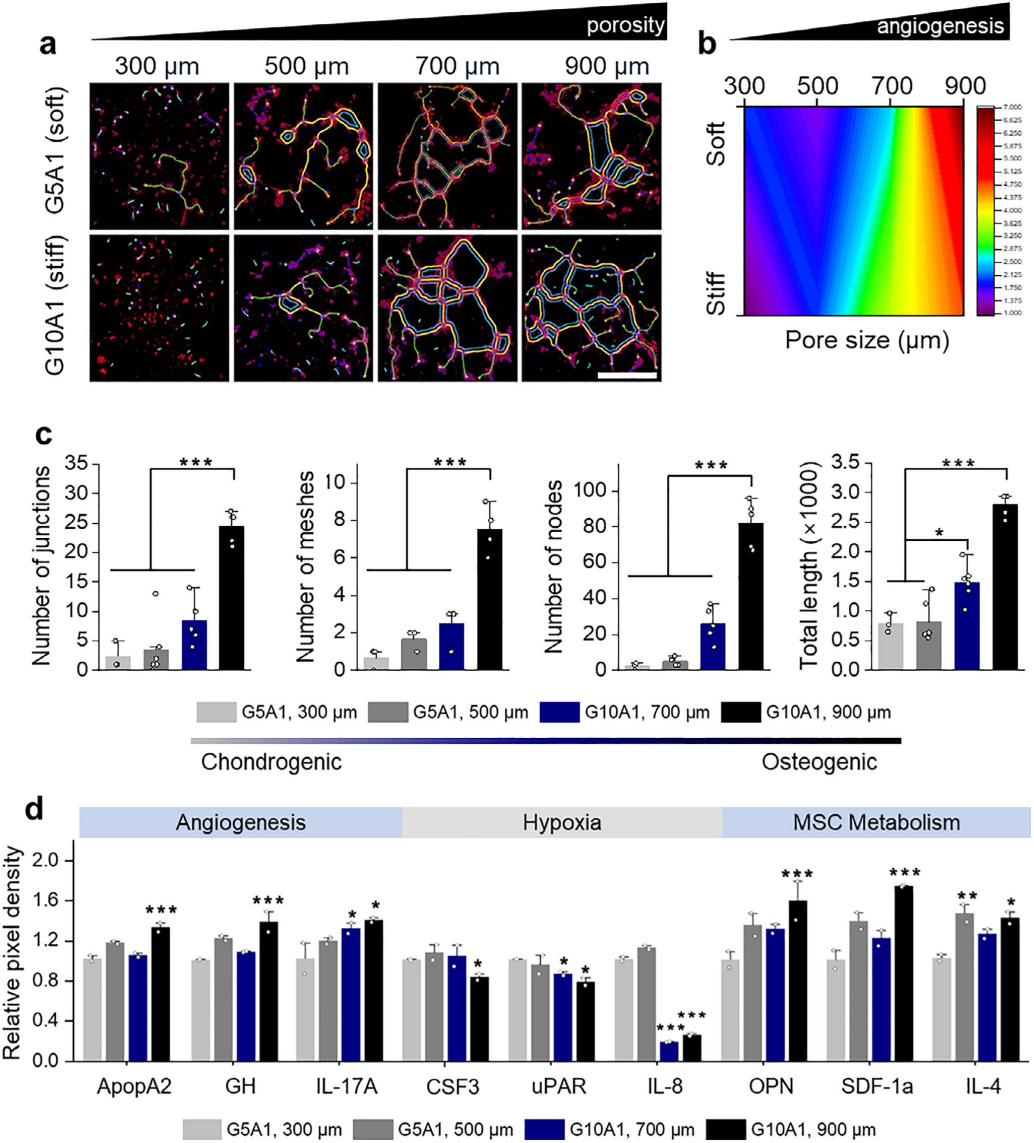
Anisotropic angiogenesis and cytokine secretion profiles. a) Representative confocal images of tube formation assay. Scale bar: 100 µm.b) Contour plot illustrating the quantitative distribution of total tube length as a function of scaffold porosity and bioink stiffness.c) Quantification of network parameters including number of junctions, number of meshes, number of nodes, and total vessel length (n ≥ 3). d) Cytokine assay of proteins secreted by hMSC spheroids cultured in the different scaffold/bioink conditions (n = 2). Statistical significance was calculated relative to G5A1, 300 µm. Data are presented as mean ± SD. Statistical significance was determined using one‐way ANOVA with Tukey's post–hoc test; ^*^ *p* < 0.05, ^#^ *p* < 0.01, ^**^ *p* < 0.005, ^***^ *p* < 0.0005.

For successful osteochondral regeneration, not only the phenotype of the newly formed tissue but also functional recovery and integration with the native tissue must occur [50]. Among the key determinants, vascularization and associated nutrient/oxygen gradients critically influence transplanted cell survival, differentiation, and integration. In subchondral bone, blood vessels deliver oxygen and nutrients essential for osteogenesis and remodeling, whereas excessive vascular invasion into cartilage disrupts its hypoxic niche, leading to fibrosis or abnormal tissue formation [51]. Therefore, replicating the native OC vascular gradient—high in bone and minimal in cartilage—is essential for long‐term function and integration. Previous studies have consistently demonstrated a positive correlation between scaffold porosity and permeability [52, 53], and have further shown that scaffolds with gradient porosity exhibit spatially heterogeneous permeability profiles depending on local porosity distribution [54]. Accordingly, the PCL scaffold designed with a porosity gradient is expected to generate spatially heterogeneous mass transport environments, which can give rise to metabolic gradients within the construct. Tube formation assays were conducted to evaluate angiogenesis by endothelial cells in both G5A1 and G10A1 hydrogels under different porosity conditions (Figure 5a; Figure S16). In both stiffness conditions, angiogenesis increased proportionally with porosity (Figure 5b). The vascular network index was quantified by the number of junctions, meshes, nodes, and total length of the formed tubes (Figure 5c). Notably, as we moved from the chondrogenic to the osteogenic layer, all vascular indices exhibited a gradual increase, suggesting a continuous transition of angiogenic activity. The degree of angiogenesis did not exhibit a sharp distinction between layers; rather, there was a smooth transition in vascularization across the layer, suggesting that fine‐tuning interfacial permeability can control vascular infiltration similar to native tissue [3].

To further explore the molecular basis of these gradients, we performed a cytokine array to evaluate cytokine production related to vascular endothelial growth, oxygen tension, and nutrient availability (Figure 5d; Figure S17). Angiogenic cytokines (ApoA2, GH, IL‐17A) were enriched in the osteogenic layer [55, 56, 57], whereas hypoxia‐associated cytokines (CSF3, uPAR, IL‐8) predominated in the cartilage layer [58, 59, 60]. Osteogenic‐related cytokines (OPN, SDF‐1α) increased progressively toward the osteogenic region, while the anti‐inflammatory cytokine IL‐4 was present in all but the uppermost layer, indicating a potential role in immune modulation and prevention of tissue damage [61, 62]. Following the cytokine analysis, we further examined whether scaffold porosity also regulates hypoxic and metabolic signaling within the constructs. As shown in Figure S18, canonical HIF target genes were differentially expressed depending on pore size. VEGF secretion, measured by cytokine analysis, was significantly increased in low‐porosity (small pore) regions, indicating enhanced hypoxia‐driven angiogenic signaling. In parallel, GLUT1 expression, quantified by qPCR, was also upregulated in low‐porosity regions, reflecting metabolic adaptation toward increased glycolytic activity under diffusion‐limited conditions. These findings indicate that our dual‐stimuli environment effectively generated spatially resolved angiogenic and hypoxic gradients. By integrating mechanical and metabolic cues, the constructs more closely mimic the native OC microenvironment, enhancing not only stem cell differentiation but also tissue functionality and long‐term stability. The capacity to control vascular and metabolic gradients holds strong translational potential for osteoarthritis repair and osteochondral defect treatment, where precise regulation of vascularization and tissue integration is essential [63].

### 2.5 Phase‐Specific Osteochondral OA Model as a Preclinical Drug Screening Platform

One major advantage of tissue‐engineered constructs is their potential use as personalized drug screening platforms [64]. We evaluated whether our osteochondral construct can be extended beyond tissue regeneration to serve as an in vitro osteoarthritis (OA) model. OA is a disease of the entire osteochondral unit, where articular cartilage and subchondral bone are structurally and functionally interconnected [65]. Biochemical and mechanical crosstalk between chondrocytes and bone cells is increasingly recognized as a key driver of OA onset and progression. Conventional cartilage‐only or bone‐only models fail to capture these interactions, limiting their physiological relevance [66]. Therefore, an in vitro OC model incorporating both tissue compartments provides a more representative platform for studying OA pathophysiology and testing therapeutic candidates.

**FIGURE 6:**
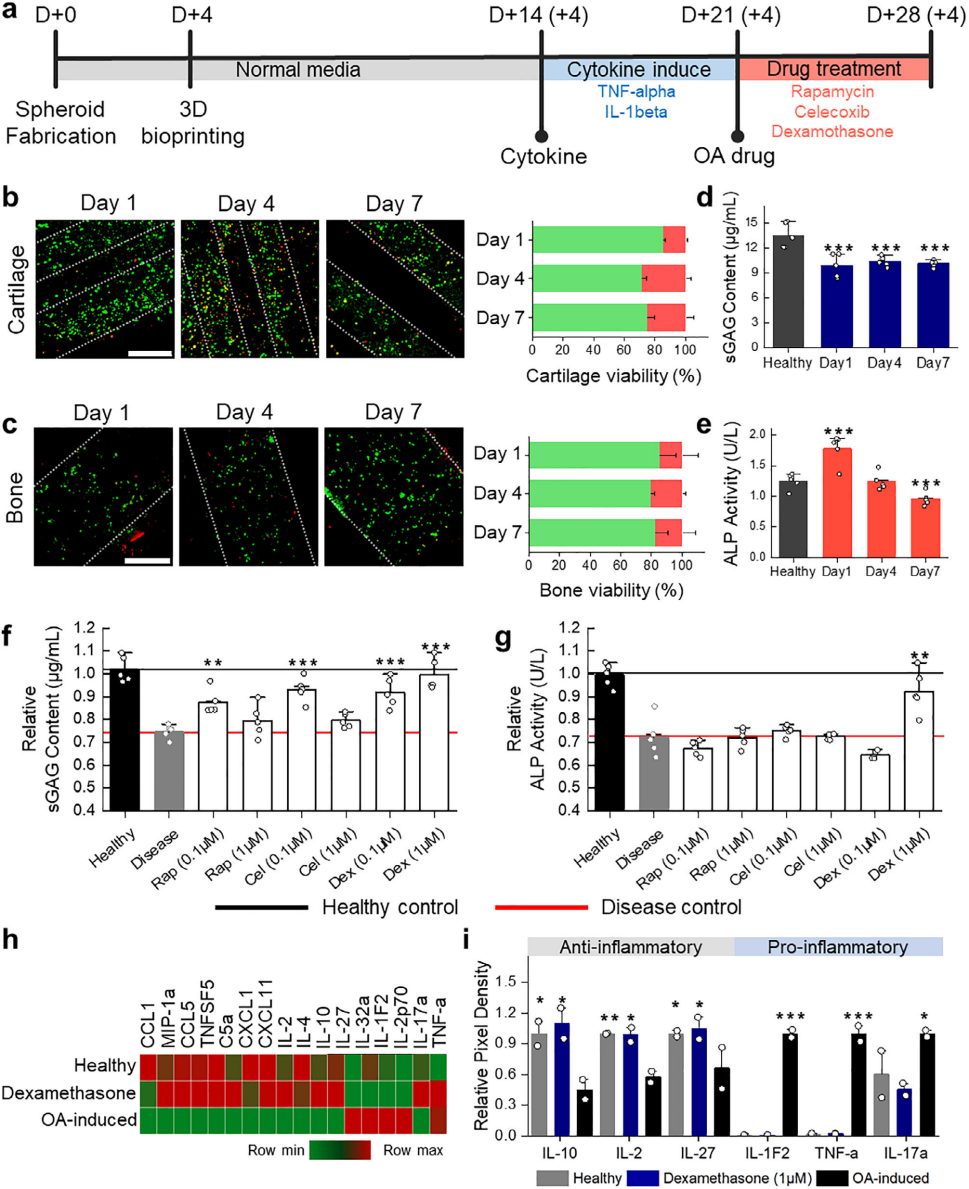
Cytokine‐induced in vitro osteoarthritis (OA) model for therapeutic screening. a) Schematic representation of overall experimental procedures. b,c) live/dead staining images and quantitative viability analyses for cartilage (b) and bone (c) regions over the culture period (n = 3). Scale bar: 500 µm. d,e) biochemical assays including sGAG content for cartilage (d) and ALP activity for bone (e) (n = 5). Statistical significance was calculated relative to healthy control. f) sGAG content in the cartilage phase for healthy control, disease control, and drug‐treated groups (n = 5). Statistical significance was calculated relative to disease control. g) ALP activity in bone phase for healthy control, disease control, and drug‐treated groups (n = 5). Statistical significance was calculated relative to disease control. h) Heatmap plot for cytokine profiling. i) quantification of anti‐inflammatory and pro‐inflammatory cytokines in healthy, oa‐induced, and dexamethasone‐treated constructs (n = 2). Data are presented as mean ± SD. Statistical significance was determined using one‐way ANOVA with Tukey's post–hoc test; ^*^ *p* < 0.05, ^#^ *p* < 0.01, ^**^ *p* < 0.005, ^***^ *p* < 0.0005.

A healthy OC unit was first generated by culturing the bioprinted construct in basal medium for two weeks. The inflammatory phenotype was then triggered by treating the construct with a combination of tumor necrosis factor‐α (TNF‐α) and interleukin‐1β (IL‐1β) for one week (Figure 6a). These cytokines are known to suppress cartilage ECM synthesis, upregulate matrix‐degrading enzymes, and promote chondrocyte apoptosis, while in bone they induce a pro‐inflammatory osteoblast phenotype, stimulate osteoclast activation, and cause abnormal remodeling [67, 68]. Following cytokine stimulation, a pronounced increase in MMP‐13, a key hallmark enzyme of osteoarthritis responsible for collagen type II degradation, was observed in the cartilage layer (Figure S19). This elevation in MMP‐13 indicates successful induction of an OA‐like catabolic state, confirming that the inflammatory treatment effectively recapitulates key pathological features of osteoarthritis in the model. Upon cytokine treatment, both cartilage and bone phases of our OC construct exhibited a progressive decrease in cell viability. Notably, the decline in cell viability was more pronounced in the cartilage phase (Figure 6b) than in the bone phase (Figure 6c). This can be attributed to the high vunerability of chondrocytes to inflammatory cytokines, leading to stronger activation of catabolic and apoptotic pathways [69]. In cartilage phase, sGAG content dropped sharply immediately after cytokine treatment (day 1) and remained at a low plateau through day 7 (Figure 6d). This pattern reflects the rapid proteoglycan depletion observed in early‐stage OA in vivo, which is driven by the elevated production of matrix‐degrading enzymes such as MMPs and ADAMTSs [70, 71]. In contrast, bone phase showed a transient increase in ALP activity at day 1 following cytokine exposure, followed by a gradual decline by day 7. (Figure 6e) This initial rise is consistent with the reactive bone remodeling process seen in early OA in vivo, where subchondral osteoblasts respond to microdamage and altered loading by temporarily elevating ALP production [72]. These phase‐specific responses closely recapitulate the distinct pathological responses of cartilage and bone during OA progression in the native joint, underscoring the physiological relevance of our model for studying disease mechanisms and evaluating targeted therapeutics.

We next tested three agents—dexamethasone, rapamycin, and celecoxib—at two different dose to probe the responsiveness to anti‐inflammatory interventions. These drugs were selected for their distinct mechanisms of action: dexamethasone suppresses upstream inflammatory transcription factors (e.g., NF‐κB, AP‐1) [73]; rapamycin inhibits mTOR to induce autophagy and exert chondroprotective, anti‐senescent effects [74]; and celecoxib selectively inhibits COX‐2 to block prostaglandin‐mediated inflammatory pathways [75]. This combination allowed us to evaluate the model's capacity to differentiate between drugs with narrow vs broad anti‐inflammatory spectra, as well as those with cartilage‐ vs bone‐specific actions. The group without cytokine treatment was designated as the healthy control, while the group treated with cytokines but without subsequent drug treatment was designated as the disease control. In cartilage phase, all three drugs increased sGAG content compared to the disease control, indicating partial restoration of ECM proteoglycan synthesis (Figure 6f). Dexamethasone significantly enhanced sGAG levels at both doses, whereas rapamycin and celecoxib showed improvement only at the lower concentration, likely due to biphasic effects where excessive pathway inhibition at higher doses suppresses anabolic activity. Strikingly, the bone phase exhibited a markedly different pattern: despite the cartilage phase showing increased recovery in most treatment groups, only 1 µm dexamethasone significantly elevated ALP activity. (Figure 6g) Our findings demonstrate that pharmacological responses in the osteochondral OA model are phase‐specific, underscoring the necessity of evaluating both cartilage and bone compartments both independently and simultaneously. This unique effect of dexamethasone can be attributed to its broad suppression of upstream inflammatory pathways (e.g., NF‐κB, AP‐1) coupled with direct promotion of osteoblast differentiation [76, 77], whereas rapamycin's mTOR inhibition suppresses osteogenic activity [78] and celecoxib's COX‐2‐specific blockade offers limited influence on bone remodeling [79]. These results parallel clinical observations that cartilage and bone often exhibit divergent therapeutic responses, reflecting their distinct biology and pathological progression in OA [69]. By recapitulating these differences in vitro, our model provides a physiologically relevant platform for phase‐specific drug evaluation, highlighting the importance of incorporating both cartilage and bone outcomes into preclinical OA therapeutic screening.

Cytokine profiling confirmed the functional relevance of these effects. As shown in Figure 6h, drug treatment shifted the cytokine expression pattern in the OA‐induced constructs toward that of the healthy condition. Specifically, anti‐inflammatory cytokines (IL‐10, IL‐2, and IL‐27), which were markedly reduced in the OA‐induced state, were restored to near‐healthy levels upon treatment. Conversely, pro‐inflammatory cytokines (IL‐1F2, TNF‐α, and IL‐17A) were substantially suppressed in both the healthy and drug‐treated groups compared to OA‐induced group (Figure 6i). Overall, our OC OA model reproduces the tissue‐specific pathology and therapeutic responses observed in vivo. It enables simultaneous evaluation of cartilage and bone outcomes, integrating structural markers (sGAG, ALP) with cytokine profiles to provide a physiologically relevant, translational platform for preclinical OA drug screening.

### 2.6 Biocompatibility and Therapeutic Efficacy Evaluation using an In Vivo Osteochondral Defect Model

To confirm the effects of osteochondral regeneration observed in vitro, we first analyzed in vivo biocompatibility using a subcutaneous implantation animal model. For this purpose, we implanted porosity only scaffold (Gradient PCL scaffold only), stiffness only scaffold (Spheroid‐encapsulating bioinks with different stiffness), and dual‐gradient scaffold (Gradient PCL scaffold printed with spheroid‐encapsulated bioinks with different stiffness) into the subcutaneous of nude mice for three weeks. Histopathological evaluation of scaffold‐implanted skin tissue showed that each scaffold group did not exhibit significant inflammatory reactions in the subcutaneous tissue and formed fibrous capsules around the scaffolds (Figure S20). GelMA‐Alginate bioinks showed a faster degradation pattern than PCL scaffolds, and inflammatory cells were observed to infiltrate into the GelMA‐Alginate hydrogel. In the PCL scaffold with/without spheroid groups, fibrous tissue and inflammatory cell infiltration were observed between the PCL fibers. However, no prominent foreign body response, such as necrosis formation or vascularization at the implantation site, was observed. In addition to this local biocompatibility assessment, the effects on systemic organs were evaluated by analyzing the histopathological responses of organs (heart, lungs, liver, kidneys, and spleen) in vivo (Figure S21). As a result, no significant toxic reactions were observed in any group at 3 weeks after implantation. However, in the cell‐loaded groups (Stiffness only, Dual‐gradient), mild to moderate hyperplasia of splenic follicles was observed. According to these results, PCL‐ or GelMA‐Alginate based scaffolds and cell loads appear to exhibit high biocompatibility and are not considered to exhibit significant toxicity locally (subcutaneously) or systemically (in vital organs).

**FIGURE 7:**
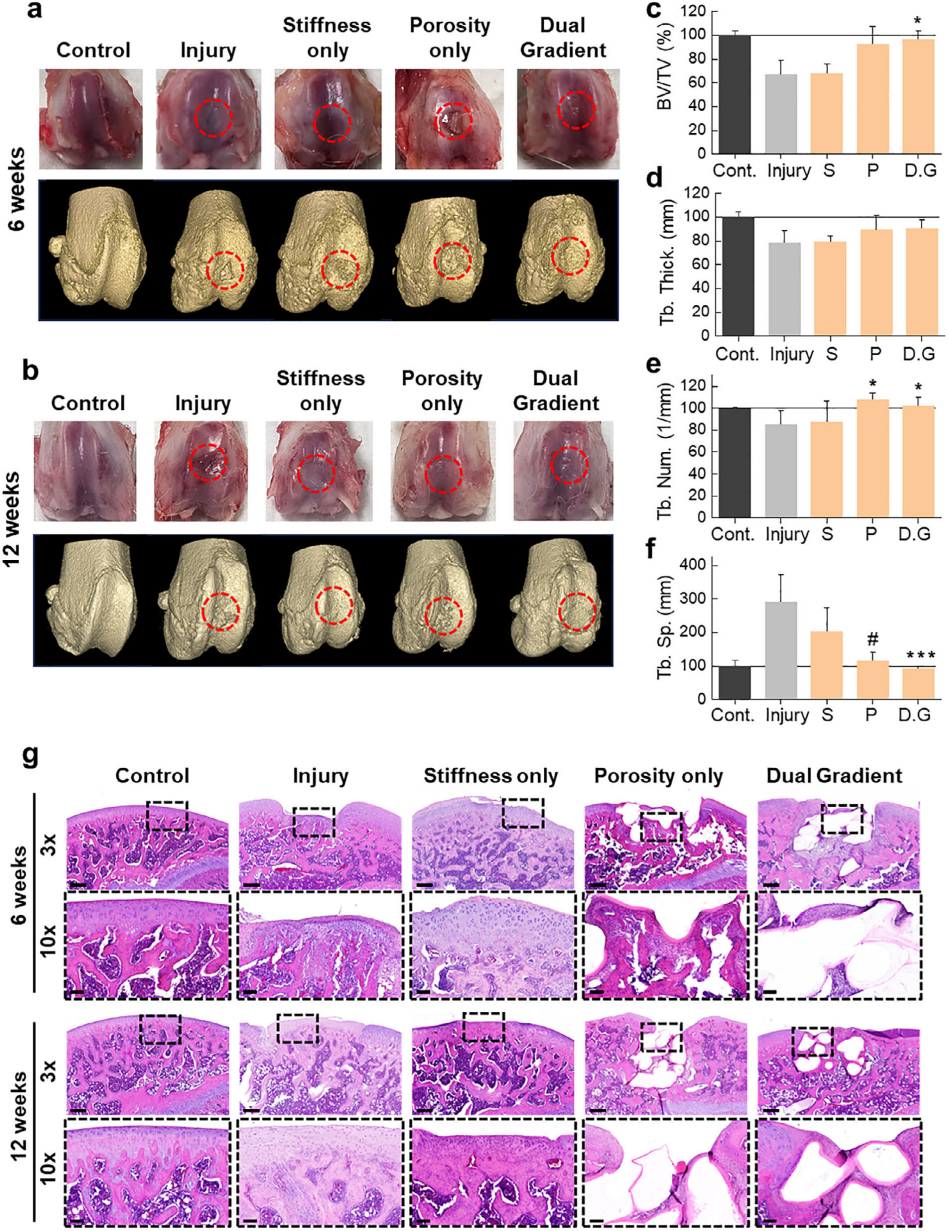
In vivo evaluation of osteochondral defect regeneration by histology and micro‐computed tomography. a,b) representative 3d reconstructed micro‐ct images of regenerative tissue with defect regions indicated by red dotted circles. (a) 6 weeks after implantation and (b) 12 weeks after implantation. c–f) quantitative micro‐ct analysis of bone volume fraction (bv/tv, %; c), trabecular thickness (tb.th, mm; d), trabecular number (tb.n, 1/mm; e) and trabecular separation (tb.sp, mm; f) in the defect region for each group (n = 3). Statistical significance was calculated relative to injury control. (*S*; Stiffness only, *P*; Porosity only, D.G; Dual gradient) g) Representative h&e‐stained sections of defect sites at 6 weeks and 12 weeks post‐implantation. Low‐magnification (3 ×) images show the overall defect region, with boxed areas enlarged at higher magnification (10 ×). scale bars: 400 µm (3 ×), 100 µm (10 ×). Data are presented as mean ± SD. Statistical significance was determined using one‐way ANOVA with Tukey's post–hoc test; ^*^ *p* < 0.05, ^#^ *p* < 0.01, ^**^ *p* < 0.005, ^***^ *p* < 0.0005.

Furthermore, we applied scaffolds loaded with cellular spheroids to a rat osteochondral defect model and analyzed their regenerative properties. To this end, a defect area with a diameter of 2–3 mm was created in the trochlear groove of the distal femur using an electric drill, and the experimental materials were implanted into the defects for 6 and 12 weeks. Macroscopically, all surgical groups (Injury, Stiffness only, Porosity only, Dual‐gradient) had defects remaining in the trochlear groove at 6 weeks (Figure 7a). However, defects in the Injury and Stiffness only group showed a relatively flat shape compared to Con group, but they did not have regenerated tissue of the same height as Con group and were found to have a clearly distinguishable boundary from the surrounding tissue. Defects in the porosity only and dual‐gradient group were found to have a grid‐like pattern remaining on the surface. In the porosity group, the defect was partially covered, while in the dual‐gradient group, most of the surface was covered with newly regenerated tissue. At week 12, defects were observed to have risen to some extent in all groups (Figure 7b). However, in the Injury and stiffness only groups, the height of the regenerated tissue differed from that of the Con group. In the porosity only group, regenerated tissue was infiltrated into the grid pattern observed at week 6, but the surface was not completely closed, whereas in the dual‐gradient group, the surface was covered with newly formed tissue. The results of the quantitative analysis of MicroCT also showed that the dual‐gradient group exhibited statistically significant differences from the Injury Only group in terms of BV/TV %, trabecular bone number, and trabecular separation (Figure 7c–f). These results suggest that the experimental group with dual‐gradient group exhibits advantages in terms of bone regeneration compared to the stiffness only or porosity only group.

Additional histopathological evaluation was performed to analyze microstructural changes in the regenerated bone tissue (Figure 7g). Histopathological analysis showed that the trochlear groove was not completely regenerated in the Injury only group at week 6, while excessive regeneration of the cartilage layer was observed in the stiffness only group. Both the porosity only and the dual‐gradient group showed that the implant surface was not completely closed, with PCL fiber cross‐sections visible in cross‐section. Interestingly, in the PCL without bioink (porosity only group), similar to the results of the subcutaneous implant, the extent of tissue infiltration into the PCL fibers was minimal, whereas in the PCL with bioink (dual‐gradient group), tissue and cellular infiltration was prominent. In the results of the 12‐week implantation, the defect layer in the Injury group tended to be replaced by eosinophilic fibrous tissue rather than normal cartilage tissue. On the other hand, the stiffness only group showed that the defect layer was replaced by cartilage tissue, but it exhibited irregularities (some overgrowth or undergrowth) on the cartilage surface compared to the Con group. In the porosity only group, as PCL gradually degraded, it was replaced by bony tissue, while the surface remained partially open. In contrast, in the dual‐gradient group, the surface was covered by regenerated tissue, and the integration between the scaffold and host tissue was observed to be maintained at a higher level compared to PCL. In particular, the cartilage around the PCL was observed to cover the PCL. These results indicate that utilizing cellular spheroid‐encapsulating bioinks with load‐bearing PCL material, which can be used as a regenerative material for osteochondral defects, not only helps the infiltration of regenerating host tissue into the PCL fibers but also aids in the tissue integration between the PCL fibers and the host tissue. Taken together, these results suggest that mechanical and metabolic dual‐gradient can promote the regeneration of bone and cartilage in situations where normal cartilaginous regeneration is difficult due to severe osteochondral defects in cartilage and bone tissue.

## 3 Conclusion

In this study, we present a developmentally inspired, multi‐material 3D‐printed osteochondral construct that integrates spatially defined mechanical and metabolic gradients to direct hMSCs fate without the need for exogenous biochemical supplementation. By embedding hMSCs as 3D spheroids and incorporating a stiffness gradient via GelMA–alginate hydrogels together with a metabolic gradient established by porous PCL frameworks, we successfully recapitulated the distinct microenvironments of articular cartilage and subchondral bone within a single, continuous construct. Our results demonstrate that mechanical stiffness could drive lineage‐specific differentiation, with softer hydrogels favoring chondrogenesis and stiffer hydrogels promoting osteogenesis. Scaffold porosity further modulated functional ECM production by influencing nutrient and oxygen diffusion, enhancing cartilage‐like matrix formation under confined conditions and osteogenic matrix deposition under high‐permeability conditions. The integration of stiffness and porosity gradients synergistically enabled spatially resolved osteochondral differentiation, resulting in a biomimetic construct with structural and functional fidelity to native tissue. Moreover, the dual‐gradient stimuli exhibit a smooth, zone‐specific functional transition in angiogenic activity. Nevertheless, further refinement of interfacial permeability may enable an even closer approximation of the highly continuous vascular transition observed in native osteochondral tissue. Beyond regeneration, we validated the construct as a physiologically relevant osteoarthritis (OA) model that preserves cartilage–bone crosstalk. Under inflammatory induction, the model reproduced compartment‐specific pathological responses, including rapid proteoglycan loss in cartilage and dysregulated bone remodeling. Phase‐specific drug screening using dexamethasone, rapamycin, and celecoxib revealed distinct therapeutic responses in each compartment, highlighting the necessity of evaluating both tissues in parallel. Furthermore, drug treatment modulated cytokine profiles toward an anti‐inflammatory state, confirming the functional relevance of observed structural recovery. In vivo implantation demonstrated effective osteochondral repair, confirming restoration of cartilage and subchondral bone architecture, as well as improved bone volume and microstructural parameters in our system. These outcomes underscore the translational potential of our dual‐function platform—as both a regeneration strategy and a preclinical drug screening model. Overall, this work establishes a robust, gradient‐engineered osteochondral platform that bridges fundamental developmental biology with advanced biofabrication. By simultaneously addressing structural, mechanical, and metabolic heterogeneity, it offers a versatile tool for functional tissue regeneration, disease modeling, and therapeutic evaluation. Future studies will focus on refining gradient resolution, extending culture longevity, and integrating patient‐derived cells to enable personalized regenerative and pharmacological applications.

## 4 Experimental Section/Methods

### 4.1 Cell Source and Culture

Primary human bone marrow–derived mesenchymal stem cells (hMSCs; PCS‐500‐012) were purchased from the American Type Culture Collection (ATCC). According to the supplier's specifications, the cells were authenticated and confirmed to be free of mycoplasma contamination. hMSCs were cultured in T175 flasks and maintained in a humidified incubator at 37°C with 5% CO_2_. The growth medium consisted of low‐glucose Dulbecco's Modified Eagle Medium (DMEM; Gibco, Invitrogen, USA) supplemented with 10% (v/v) fetal bovine serum (FBS; Gibco, USA), 1% penicillin–streptomycin (PS; Fisher Scientific, USA), and recombinant human FGF basic/FGF2/bFGF (146 aa) protein (50 µL per 50 mL of culture medium; Bio‐Techne 233‐FB/CF, USA). The medium was replaced every three days, and cells were passaged at ∼90% confluence using 0.025% trypsin (Gibco). Cells at passages 4–7 were used for all experiments.

### 4.2 hMSC Spheroid Formation

hMSC spheroids were generated using AggreWell400 plates (24‐well, inverted pyramid‐shaped microwells; Stem Cell Technologies) following the manufacturer's protocol. Each well was first treated with 500 µL of Anti‐Adherence Rinsing Solution (Stem Cell Technologies) to create an ultralow attachment surface, centrifuged at 3000 rpm for 10 min, and rinsed twice with phosphate‐buffered saline (PBS). hMSCs were detached, counted, and seeded into the plates at 1200 microwells per well. Spheroids were cultured in low‐glucose DMEM supplemented with 0.1% FBS and 1% penicillin–streptomycin at 37°C in a humidified 5% CO_2_ atmosphere. The optimized condition consisted of 500 cells per microwell (60 000 cells per well) cultured for 4 days.

### 4.3 Spheroid Morphology Characterization

Spheroid formation was evaluated using CellTracker Green CMFDA (Invitrogen) labeling. The dye was prepared as a 10 mm stock solution in DMSO and diluted 1:1000 in phosphate‐buffered saline (PBS) to prepare the working solution. hMSCs were detached, incubated in the working solution for 30 min at 37°C, centrifuged to remove residual dye, and resuspended in fresh culture medium. Labeled cells were seeded into AggreWell400 plates to form spheroids. Confocal imaging was performed using a STELLARIS 5 microscope (Leica). Morphometric parameters, including aspect ratio, circularity, and diameter, were quantified at days 1, 4, and 7 using ImageJ software.

### 4.4 Synthesis of Gelatin Methacrylate (GelMA)

10% (w/v) gelatin from porcine skin (G1890, Sigma–Aldrich, USA) was dissolved in 100 mL PBS at 50°C for 1 h. Methacrylic anhydride (8 mL; 276685, Sigma–Aldrich, USA) was added dropwise to the gelatin solution and stirred at 50°C for 2 h. An additional 100 mL of preheated PBS (50°C) was then added, and the mixture was dialyzed against ultrapure water at 40°C for 5 days (molecular weight cut‐off 12–14 kDa; 888–11530, Spectrum Chemical Mfg. Corp., USA), with water changes twice daily and inversion of the dialysis bag to ensure homogeneity. The dialyzed solution was filtered through a 0.22 µm vacuum filtration unit, frozen at −80°C, and lyophilized for 5 days to obtain GelMA in powder form.

### 4.5 Chemical Characterization of GelMA

The degree of methacrylation (DoM) of GelMA was determined to be 61.3%, as quantified by ^1^H nuclear magnetic resonance (NMR) analysis. ^1^H NMR spectra were acquired using a 400 MHz NMR Spectrometer (Advance 400, Brucker, Germany). Before analysis, 2 mg of GelMA and gelatin were dissolved in 1.5 mL of deuterium oxide (D2O; Sigma–Aldrich, USA) before measurement. The DoM was calculated by comparing the lysine methylene proton signals at δ = 2.9 ppm in GelMA with those of pristine gelatin using the following Equation (1).

Equation (1):

(1) $$\begin{array}{ccc} & & \mathrm{Degree}\;\mathrm{of}\;\mathrm{Metharylation}\;\;\left(\mathrm{DOM},\%\right)\\ & & \;=1-\frac{\left(\mathrm{lysine}\;\mathrm{methylene}\;\mathrm{proton}\;\mathrm{signal}\;\mathrm{of}\;\mathrm{GelMA}\right)\;\;}{\left(\mathrm{lysine}\;\mathrm{methylene}\;\mathrm{signal}\;\mathrm{of}\;\mathrm{gelatin}\right)}\times 100 \end{array}$$

### 4.6 Mechanical and Rheological Characterization

All rheological measurements were performed using a rheometer (Discovery HR‐2, TA Instruments, USA) equipped with a 20 mm diameter parallel‐plate geometry (TA Instruments, 115796) and a 200 µm gap. Viscosity was evaluated by conducting a shear rate sweep from 0.1 to 100 s^−^^1^ at 25°C, as well as by monitoring viscosity changes between 4 and 37°C. The complex modulus was determined via an oscillatory amplitude sweep from 1.0% to 100% strain at a frequency of 1.0 Hz, performed at 25°C. Compressive strength was measured using cylindrical hydrogel samples (8 mm diameter, 3 mm thickness). Stress relaxation behavior was assessed by applying a 10% uniaxial compressive strain and recording the decay of stress over a 600 s period at 25°C. Mechanical measurement of PCL frame was performed using universal testing machine (Instron 3344, Instron Corp, MA, USA) at a compression rate of 1mm/min.

### 4.7 Immunofluorescence Analysis

Hydrogels were washed twice with PBS for 30 min each and fixed in 4% paraformaldehyde for 50 min at room temperature. Samples were permeabilized with 0.1% Triton X‐100 in PBS for 1 h and blocked with 1% bovine serum albumin (BSA) for 1 h at room temperature. Following blocking, samples were incubated overnight at 4°C with the following primary antibodies: anti‐SOX9 (1:200, Abcam, ab26414), anti‐COL2A1 (1:200, Abcam, ab34712), anti‐RUNX2 (1:200, Abcam, ab192256), anti‐COL1A1 (1:200, Abcam, ab254113) and anti‐YAP (1:250, Santa Cruz Biotechnology, sc‐101199). After two PBS washes, samples were blocked again with PBS containing 1% BSA and 2% goat serum for 1 h at room temperature, followed by incubation with Alexa Fluor–conjugated secondary antibodies (1:200, Invitrogen) for 90 min at 37°C. Nuclei were counterstained with DAPI (1 µg/mL, Invitrogen) for 10 min. Samples were washed twice with PBS and imaged using a confocal laser scanning microscope (STELLARIS 5, Leica). Quantitative image analysis was performed using ImageJ.

### 4.8 Alcian Blue and Alizarin Red Staining

To evaluate the influence of pore size on chondrogenic and osteogenic induction, glycosaminoglycan (GAG) deposition and calcium mineralization were assessed using Alcian blue and Alizarin red staining, respectively. hMSC spheroids (500 cells/spheroid) were embedded in hydrogels with identical compositions and culture conditions, and subsequently cultured within PCL scaffolds with different pore sizes. To ensure lineage‐appropriate mechanical environments, G5A1 hydrogels were used for chondrogenic ECM evaluation, while G10A1 hydrogels were used for osteogenic ECM evaluation. Specifically, constructs cultured under the G5A1 condition were stained with Alcian Blue (TMS‐010, Sigma–Aldrich) to visualize glycosaminoglycan (sGAG) deposition, whereas constructs cultured under the G10A1 condition were stained with Alizarin Red S (TMS‐008, Sigma–Aldrich) to detect calcium mineralization. In both cases, only the scaffold pore size was varied, while hydrogel composition, spheroid configuration, culture medium, and culture duration were kept constant. After 14 days of culture in standard growth medium, samples were washed twice with PBS and incubated with the respective staining solution for 1 h at room temperature. The staining solution was then aspirated, and constructs were thoroughly rinsed with deionized water until no excess dye remained. Stained samples were imaged using an optical microscope (TS100, Nikon).

### 4.9 Cytokine Array

Cytokine profiling was conducted using the Proteome Profiler Human Cytokine Array Kit XL for metabolic gradient studies and the standard Proteome Profiler Human Cytokine Array Kit for the drug‐screening platform (R&D Systems), in accordance with the manufacturer's protocols. Chemiluminescent signals were detected using a CCD imager (Amersham ImageQuant 800, Cytiva) and analyzed quantitatively. Conditioned media were collected at 48 h, immediately frozen at −80°C, and processed for analysis. Densitometric measurements were performed manually using ImageJ software. The integrated density of each cytokine spot was normalized to the corresponding positive control spot and expressed as a percentage. For each cytokine, two replicate spots were analyzed, and mean values ± standard deviations (SD) were calculated.

### 4.10 Tube Formation Assay

Human primary umbilical vein endothelial cells (HUVEC; PCS‐100‐010) was purchased from the American Type Culture Collection (ATCC). According to the supplier's specifications, the cells were authenticated and confirmed to be free of mycoplasma contamination. Each well of 24 well plate was loaded with 150 µL of Geltrex and incubated at 37°C for 30 min to allow gelation. HUVECs were then seeded at a density of 9 × 10^4^ cells/well in 0.5 mL of conditioned media. After 24 h of incubation, phase‐contrast images were captured using a fluorescence microscope (Nikon Ti2). Quantitative analysis of capillary‐like structures—including total tube length, number of junctions, number of meshes, and mesh area—was performed using the *Angiogenesis Analyzer* plugin for ImageJ software. All assays were performed more than triplicate.

### 4.11 sGAG/ALP Assay

sGAG and ALP assays were performed using the Sulfated Glycosaminoglycan Quantification Kit (Amsbio) and the PicoSens Alkaline Phosphatase (ALP) Activity Assay Kit (Biomax), respectively, following the manufacturers’ protocols. Briefly, for sGAG quantification, 0.1 mL of each sample or standard was mixed with 0.1 mL of dye reagent in a 96‐well plate. Absorbance was measured at 515–530 nm using a Tecan microplate reader (Spark 10 m). For ALP assay, 120 µL of standards or 80 µL of samples were added to wells, followed by 50 µL of 5 mm pNPP substrate. After incubation at 37°C for 60 min, 20 µL of stop solution was added, and absorbance was measured at 405 nm using a Tecan microplate reader (Spark 10 m). Appropriate background controls were included for media and reagents.

### 4.12 Bioink Preparation

Bioinks with 10% GelMA and 1% Alginate (G10A1) and GelMA 5% with Algiante 1% (G5A1) were prepared as following procedure. Desired w/v % of GelMA and 0.5 w/v % of Irgacure 2959 (410896, Sigma–Aldrich, USA) was dissolved in phosphate‐buffered saline (PBS). 1 w/v% of sodium alginate (W201502, Sigma–Aldrich, USA) were subsequently mixed with the GelMA solution to obtain a homogeneous hydrogel precursor solution. Prior to bioprinting, hMSCs were assembled into spheroids (500 cells per spheroid) and cultured for 4 days. The spheroids were harvested by gentle pipetting with phosphate‐buffered saline (PBS) and collected by centrifugation at 100 rcf for 5 min. The collected spheroids were then resuspended in the hydrogel precursor solution to prepare spheroid‐encapsulating bioinks. The final cell density was adjusted to 6 × 10^6^ cells/mL, corresponding to 1200 spheroids/mL of bio‐ink.

### 4.13 3D Bioprinting

3D bioprinting was performed using a BIO X bioprinter (CELLINK, Sweden) equipped with three printhead slots. The printing geometry was designed in advance, and the corresponding G‐code file was generated based on the construct design.

Hydrogel bioink printing was carried out using a temperature‐controlled printhead (CELLINK, Sweden). To maintain stable rheological properties during extrusion, both the printhead and the print bed temperatures were maintained at 20°C. Bioinks (G5A1 and G10A1) were extruded through a 25‐gauge sterile needle (CELLINK, Sweden) under an extrusion pressure ranging from 60 to 100 kPa. The printing speed for hydrogel bioinks was set to 3 mm/s. For fabrication of the porosity gradient framework, polycaprolactone (PCL; Mw ≈ 50 000; Polysciences, USA) was printed using a thermoplastic printhead (CELLINK, Sweden). The printhead temperature was set to 120°C, and a thermoplastic nozzle with an inner diameter of 0.4 mm was used. PCL extrusion was performed at a pressure of 180 kPa, with a printing speed of 2 mm/s. The layer height was set to 80% of the nozzle diameter for each material. After printing, the printed constructs were photocrosslinked under UV irradiation at an intensity of 15 mW/cm^2^ for 3 min. Subsequently, ionic crosslinking was performed by immersing the constructs in a 50 mm Calcium chloride dihydrate (CaCl_2_; Sigma–Aldrich, USA) solution for 5 min, followed by thorough washing with PBS. The printed constructs were then cultured for 14 days in low‐glucose Dulbecco's Modified Eagle Medium (DMEM; Gibco, Invitrogen, USA) supplemented with 10% (v/v) fetal bovine serum (FBS; Gibco, USA) and 1% penicillin–streptomycin (PS; Fisher Scientific, USA).

### 4.14 Cytokine‐Based Osteoarthritis Induction

To investigate cartilage–bone crosstalk in osteoarthritis, full‐thickness osteochondral constructs were 3D‐printed in 6‐well plates and matured for 14 days. Constructs without cytokine exposure were defined as the “healthy” condition. Osteoarthritis was induced by treating constructs with pro‐inflammatory cytokines for 7 days. Specifically, TNF‐α (Gibco) and IL‐1β (Gibco) were diluted to a final concentration of 10 ng/mL each in culture medium containing 1% FBS and 1% P/S. Cytokine‐containing media were replaced by half every 2 days. For live/dead assays, the upper layer was defined as the cartilage region and the bottom layer as the bone region. For all other assays, supernatants from full‐thickness constructs were collected. Culture supernatants were stored at −80°C prior to analysis.

### 4.15 Validation of the Osteochondral Construct (OC) Model as a Drug‐Screening Tool

The response of the OC model to various drugs and concentrations was assessed. Dexamethasone (1 µm, 10 µm), celecoxib (10 ng/mL, 500 ng/mL; 0.1 µm), and rapamycin (10 nm, 1 µm) were tested. All drugs were dissolved in DMSO and stored at −80°C until use. Working solutions were prepared by diluting the drugs to the indicated concentrations in culture medium containing 1% FBS and 1% P/S. Drug‐containing media were replaced by half every 2 days for a total treatment duration of 7 days. Post‐treatment, drug responses were evaluated by ALP activity assay, sGAG quantification, and cytokine array analysis.

### 4.16 Statistical Analysis

Statistical analyses were performed using at least three independent samples unless otherwise noted. Data are presented as mean ± standard deviation (SD), with N indicating the number of experimental replicates. One‐way ANOVA followed by Tukey's Honestly Significant Difference (HSD) post–hoc test was applied to determine statistical significance. Error bars represent SD, and results were derived from a minimum of three independent experiments. Differences were considered statistically significant at *p* < 0.05 (^*^ *p* < 0.05, ^#^ *p* < 0.01, ^**^ *p* < 0.005, ^***^ *p* < 0.0005).

### 4.17 qRT‐PCR

Human bone marrow derived MSCs (hBMCs), either as single‐cell suspensions or pre‐formed spheroids, were encapsulated within 10% (w/v) GelMA hydrogels at a final cell density of 6 × 10^6 cells/mL. The constructs were cultured for 7 days in low‐glucose DMEM supplemented with 1% FBS under standard conditions. Following culture, the samples were washed twice with PBS (15 min each wash) and subsequently prepared by freezing the cell component at −80°C. Total RNA was isolated from the samples using the NucleoSpin RNA Plus RNA extraction kit (Macherey‐Nagel, USA) according to the manufacturer's instructions. The concentration of separated RNA was measured, and cDNA was synthesized using the ReverTra Ace qPCR RT Kit (Toyobo, Japan). The synthesized cDNA was diluted and then subjected to real‐time PCR for 45 cycles using Thunderbird Next Sybr qPCR Mix (Toyobo, Japan) on a real‐time PCR machine (CFX384, Bio‐Rad, USA). The primers were categorized into stemness and house keeping genes (Table S1). Based on the RefFinder software verification results, the average values of glyceraldehyde‐3‐phosphate dehydrogenase (GAPDH), β‐actin, and 18S ribosomal RNA (18S rRNA) were used as reference genes. Single cells encapsulated in hydrogel were used as the control, and the results were expressed as fold change in threshod cycle (Ct) using the 2^−ΔΔCT^ method.

### 4.18 In Vitro Accelerated Degradation Assay

An in vitro accelerated degradation assay was performed to compare the degradation behavior of the hydrogel bioinks and the PCL scaffold. Hydrogel samples were prepared as cylindrical disks with a diameter of 8 mm and a thickness of 3 mm, while PCL scaffolds were 3D‐printed with gradient porosity into disks of 8 mm diameter and 1.5 mm thickness.For accelerated degradation, collagenase type I (Gibco, USA) was dissolved in PBS to prepare a solution with an enzyme activity of 20 U mL^−^^1^. All samples were fully immersed in the collagenase‐containing solution and incubated at 37°C. After the designated incubation period, samples were collected, thoroughly rinsed with PBS, and freeze‐dried.Degradation was quantified by measuring the dry mass loss, and the remaining mass was normalized to the initial dry weight. All experiments were performed with n = 3 independent samples.

### 4.19 ELISA Assay

The conditioned media from 3D osteochondral constructs culture media before and after cytokine induction were collected and stored at −80°C before use. The secretion of

MMP‐13 was detected using sandwich ELISA kit (EHMMP13, Invitrogen, USA), following the protocol provided by the manufacturers. OD values were scanned at 450 nm using a Tecan microplate reader (Spark 10 m).

### 4.20 In Vivo Experiment

Two types of in vivo experiments were conducted to evaluate biocompatibility and osteochondral regeneration capacity using a mouse subcutaneous implantation model and a rat osteochondral defect model, respectively. All animal experiments were conducted under the approval of the Institutional Animal Care and Use Committee (IACUC) at Korea University (approval number: KUIACUC‐2024‐0074). For the subcutaneous implantation study, twelve Nude mice (male, 7 weeks) were used and categorized into four groups as follows: Control, PCL scaffold only (Porosity only), Spheroids‐encapsulating GelMA scaffold with different stiffenss (Stiffness only), PCL scaffold with spheroid‐encapsulated bioinks (Dual‐gradient). In Stiffness only and dual gradient group, G5A1 hydrogel were used for cartilage layer and G10A1 layer for subchondral bone part. Before surgery, antibiotics (enrofloxacin, 5 mg/kg) and analgesics (carprofen, 5 mg/kg) were administered to the animals to prevent infection and excessive pain. Inhalation anesthesia was induced using 4% isoflurane mixed with oxygen, and anesthesia was maintained with 1.5–2.5% isoflurane. The dorsal skin was disinfected with povidone/70% alcohol and incised with Metzenbaum scissors to a length of approximately 1 cm. The incised skin was bluntly separated, and a 6 mm diameter circular sample was transplanted. Three weeks after implantation, the animals were euthanized using carbon dioxide (CO_2_), and their skin and major organs (heart, lungs, liver, kidneys, spleen) were collected and fixed in 4% neutralized buffered formalin (NBF, BBC).

For the osteochondral defect model, 24 Sprague‐Dawley rats (male, 7 weeks) were used and categorized into four groups as follows: Injury, stiffness only, porosity only. dual‐gradient group. The control group was set as the opposite leg that did not develop osteochondral defects. Pre‐medication (analgesia, antibiotics) and anesthesia were administered ㄴ in the same manner as in the subcutaneous study. After making a skin incision on the lateral surface of the femur, the muscle and joint capsule were incised to expose the trochlear groove of the distal femur. After creating a 2–3 mm diameter defect using an electric drill, a 2 mm scaffold was implanted into the osteochondral defect. After transplantation, the joint capsule and skin were sutured using absorbable sutures and non‐absorbable sutures/surgical clips, respectively. The transplanted animals were euthanized 6 and 12 weeks after transplantation, and the bones were fixed in 10% NBF.

### 4.21 MicroCT Evaluation

The distal femurs from all experimental group were examined using microcomputed tomography (µ‐CT) with a Quantum GX2 microCT (PerkinElmer, USA). Quantitative analysis was performed using CTAn software (Bruker, Germany). The region of interest (ROI) was set as a cylinder with a diameter of 3 mm and a height of 3 mm. Morphometric parameters included percent bone volume ratio (bone volume/tissue volume (BV/TV, %), trabecular bone thickness (mm), trabecular bone number (Tb.Num., 1/mm), and trabecular bone separation (mm).

### 4.22 Histopathological Evaluation

For skin and major organ samples, tissue samples fixed with NBF were processed using standard paraffin tissue processing methods (dehydration and paraffin infiltration). Paraffin Sections 4–5 µm thick were prepared and hematoxylin and eosin (HE) staining were performed. For bone samples, decalcification was performed using Rapid Cal‐immuno decalcification solution before creating paraffin blocks, followed by tissue processing. The stained slides were fixed in mounting medium and scanned using a histopathological slide scanner (Motic microscope, MoticEasyScan One, USA), and images were obtained and analyzed using QuPath software.

## Conflicts of Interest

The authors declare no conflict of interest.

## Supporting information




**Supporting File 1**: advs74451‐sup‐0001‐SuppMat.docx.

**Supporting File 2**: advs74451‐sup‐0002‐TableS1.docx.

## Data Availability

The data that support the findings of this study are available in the supplementary material of this article.
