# Supplementary material for: Developmentally Inspired, Mechanical–Metabolic Dual Gradient Osteochondral Constructs Bridging Regeneration and Therapeutic Screening
Source: Adv Sci (Weinh). 2026 Mar 3;13(24):e16602. doi: 10.1002/advs.202516602 (PMC13116013; doi:10.1002/advs.202516602)
Supplement: Supplementary file 1 — Supporting File 1: advs74451‐sup‐0001‐SuppMat.docx. [file ADVS-13-e16602-s002.docx]

Supporting Information

**Developmentally Inspired, Mechanical–Metabolic Dual Gradient**

**Osteochondral Constructs Bridging Regeneration and Therapeutic Screening**

*Yurim Choi, Wonjun Jang, Raehui Kang, Ulziituya Batjargal, Jihyeon Song, Soojin Park, Min-Seok Kim, Hyobum Cho, Junhyung Kim, Yu Shrike Zhang, Han-Jun Kim^*^, Junmin Lee^*^*


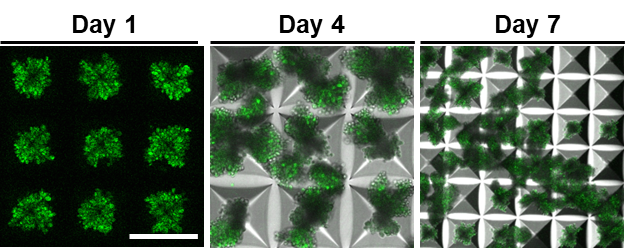


**Figure S1. Representative fluorescent images showing morphological changes of hMSC spheroids formed from 1000 cells/microwell over a 7-day culture period.** Scale bar: 400 µm.

**
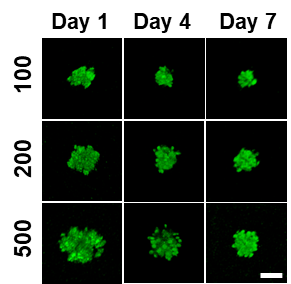
**

**Figure S2. Representative fluorescent images of hMSCs spheroids cultured in microwells for morphological analysis.** Spheroids were fluorescently labeled with CellTracker green. Scale bar: 50 µm.

**
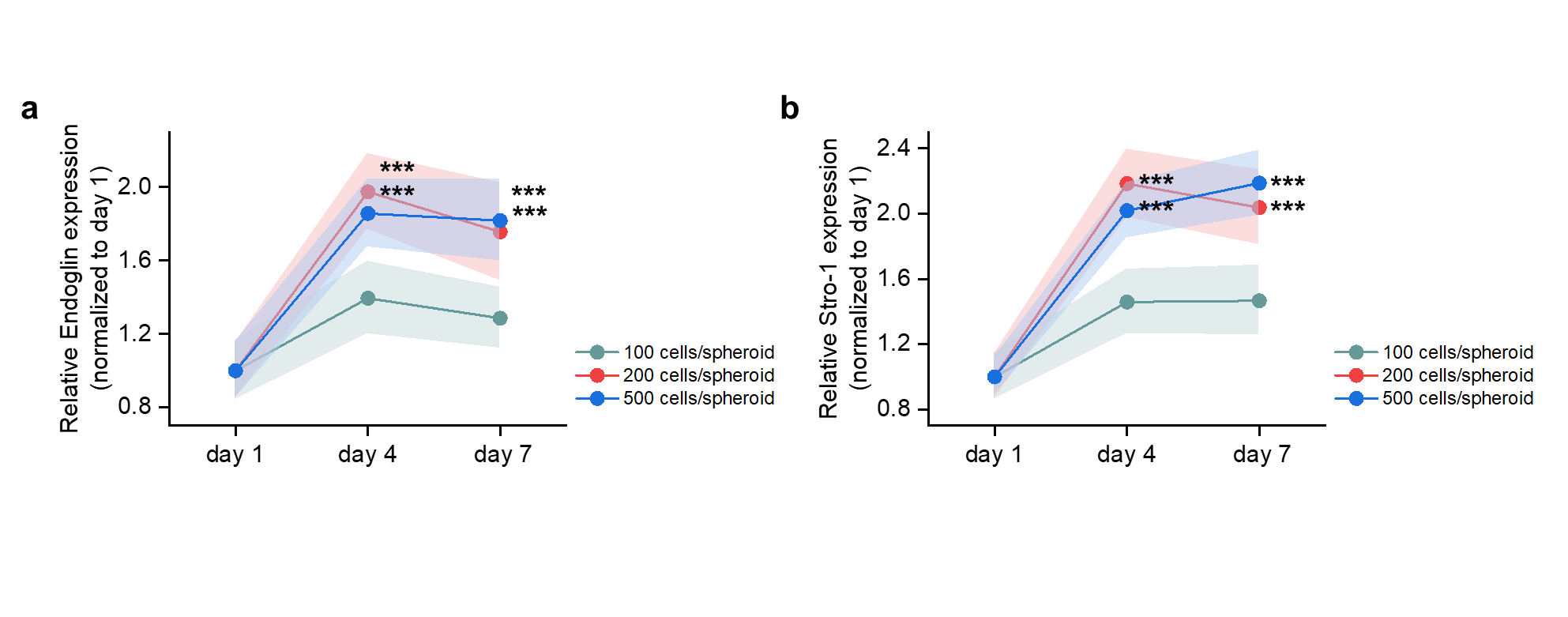
Figure S3. Temporal changes in marker expression according to spheroid cell number.**

(a) Quantification of relative endoglin expression on days 1, 4, and 7 for spheroids containing 100, 200, or 500 cells per spheroid (b) Quantification of relative Stro-1 expression expression on days 1, 4, and 7 for spheroids containing 100, 200, or 500 cells per spheroid.


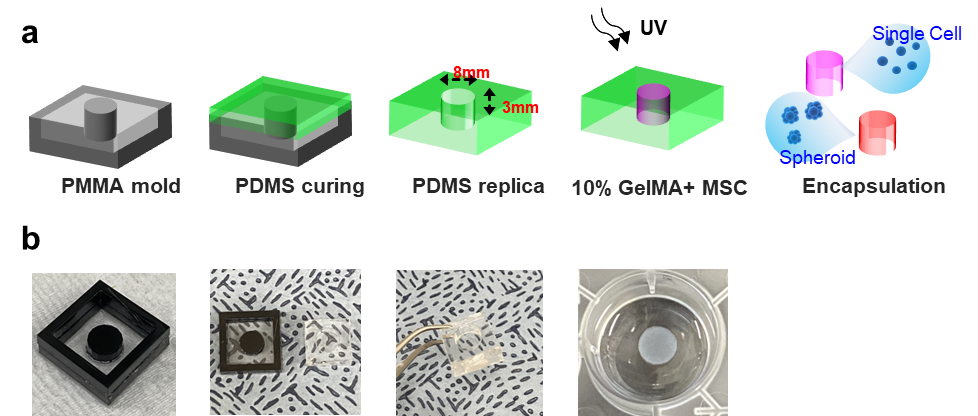


**Figure S4. Fabrication of PDMS molds for single-cell and spheroid encapsulation.** Schematic (a) and photographs (b) showing (from left to right): PMMA mold preparation, PDMS curing, PDMS replica with cylindrical well (diameter: 5 mm, height: 3 mm), loading of 10% GelMA containing hMSCs, and encapsulation of either single-cell suspensions or preformed spheroids.


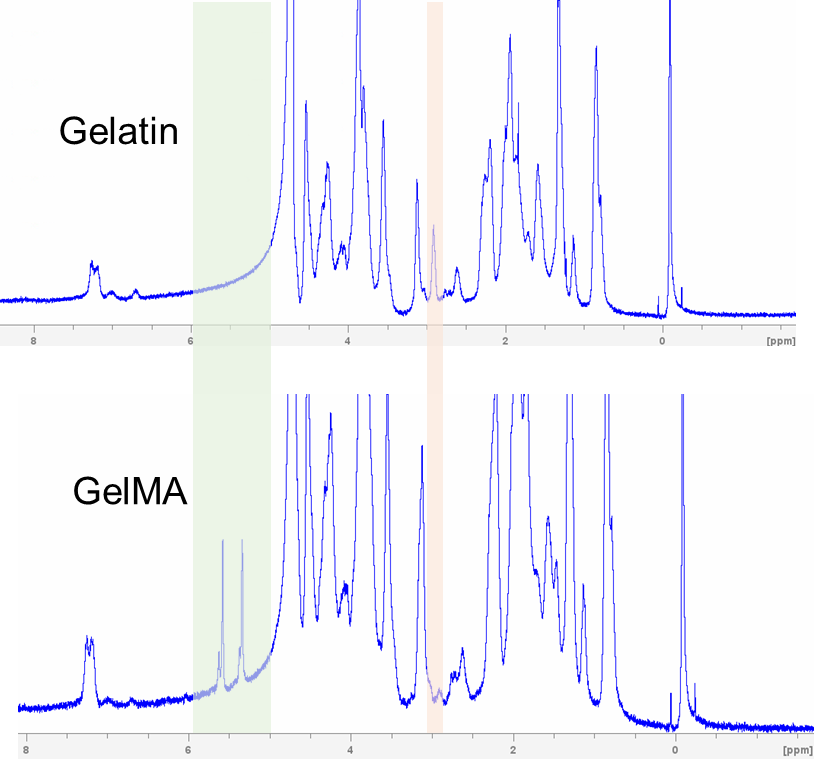


**Figure S5. 1H NMR spectra of gelatin (top) and GelMA (bottom).** Successful methacrylation of gelatin was confirmed by the appearance of new peaks at ~5.3–5.7 ppm corresponding to vinyl protons (green highlight) and at ~1.8–2.0 ppm corresponding to methyl groups of methacrylate (orange highlight).

**
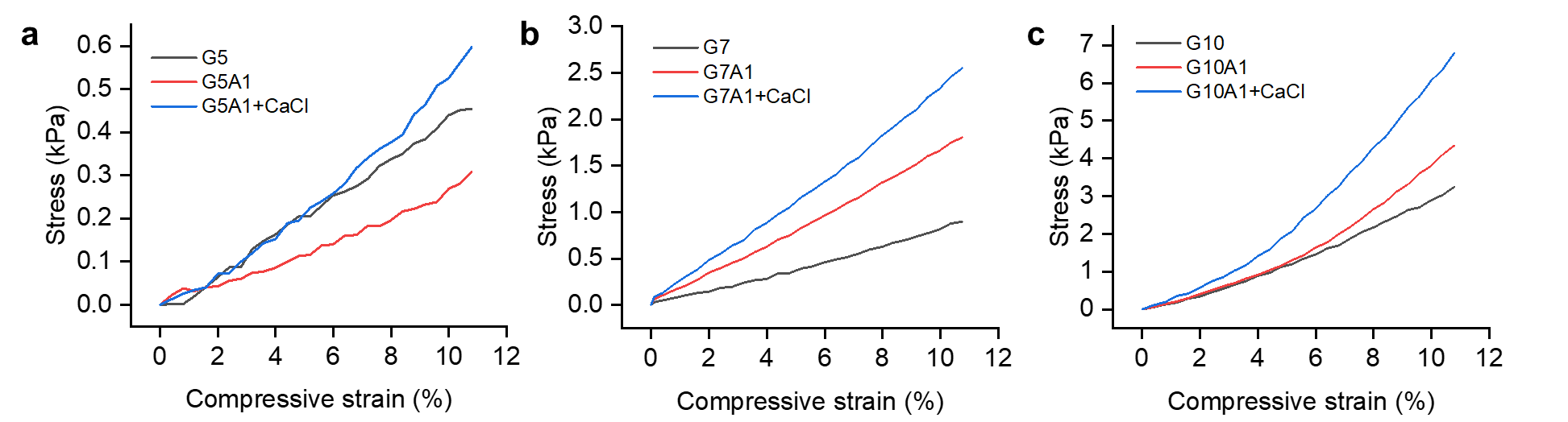
Figure S6.** **Representative compressive stress–strain curves of hydrogel samples with different compositions and crosslinking conditions.** (a) GelMA 5% with and without alginate and CaCl₂ treatment (b) GelMA 7% with and without alginate and CaCl₂ treatment (c) GelMA 10% with and without alginate and CaCl₂ treatment.


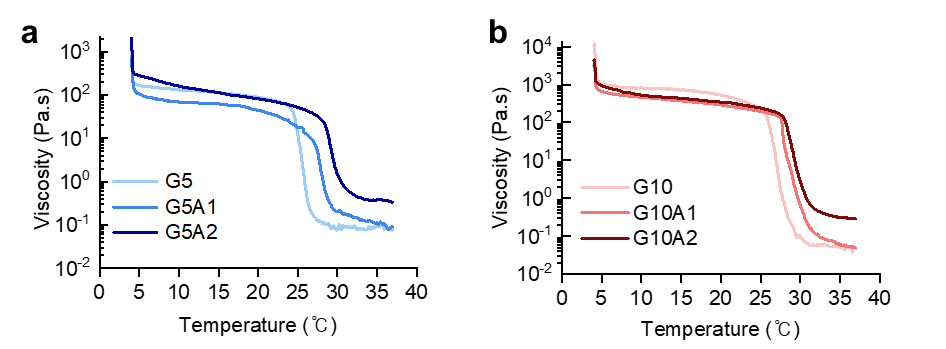


**Figure S7. Sol–gel transition temperature of GelMA-based bioinks with varying alginate content, determined by temperature sweep rheometry.** (a) Rheological behavior for G5, G5A1, and G5A2 formulations. (b) Rheological behavior for G10, G10A1, and G10A2 formulations. The addition of alginate increased the sol–gel transition temperature in both GelMA concentrations.


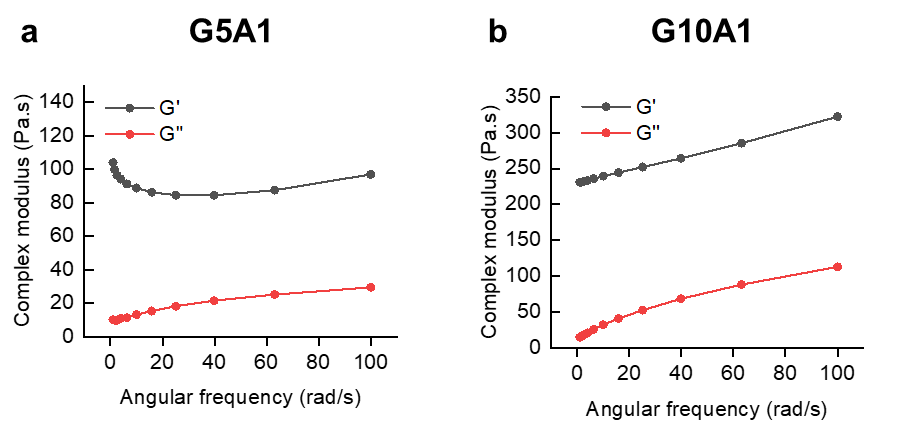


**Figure S8. Frequency sweep test of bioinks.** (a) Rheological behavior for G5, G5A1, and G5A2 formulations. (b) Rheological behavior for G10, G10A1, and G10A2 formulations In both bioinks, G′ exceeded G″ at all tested frequencies, indicating a predominantly elastic behavior.


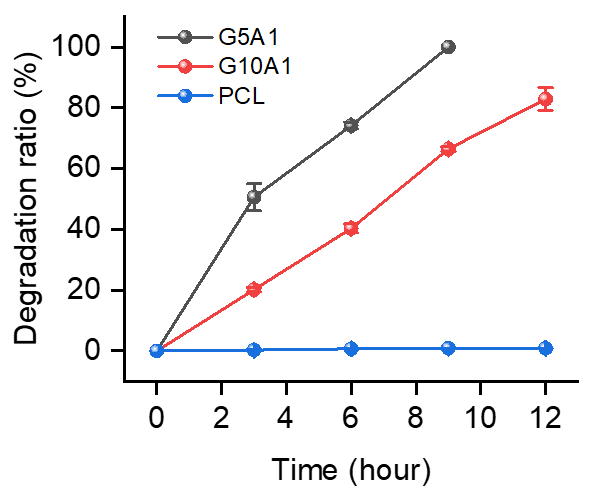


**Figure S9. Degradation kinetics of G5A1 (soft) hydrogel, G10A1 (stiff) hydrogel, and PCL framework.**


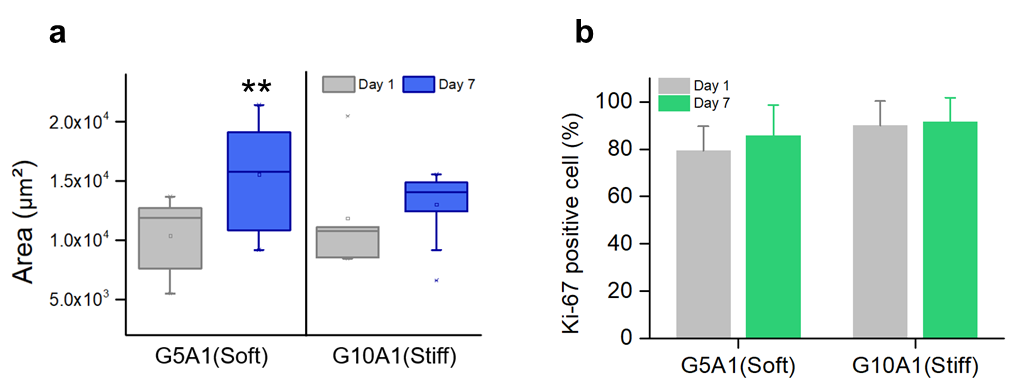


**Figure S10. Quantification of area and Ki-67 positive cells of hMSC spheroids encapsulated in bioinks.** (a) Quantification of spheroid area on days 1 and 7, showing a significant increase in the soft hydrogel (**p < 0.01) but no change in the stiff hydrogel. (b) Percentage of Ki-67–positive cells on days 1 and 7, indicating sustained proliferative activity in both hydrogel types. Data are presented as mean ± SD (n = 3). Statistical analysis was performed using two-way ANOVA with Tukey’s post hoc test.


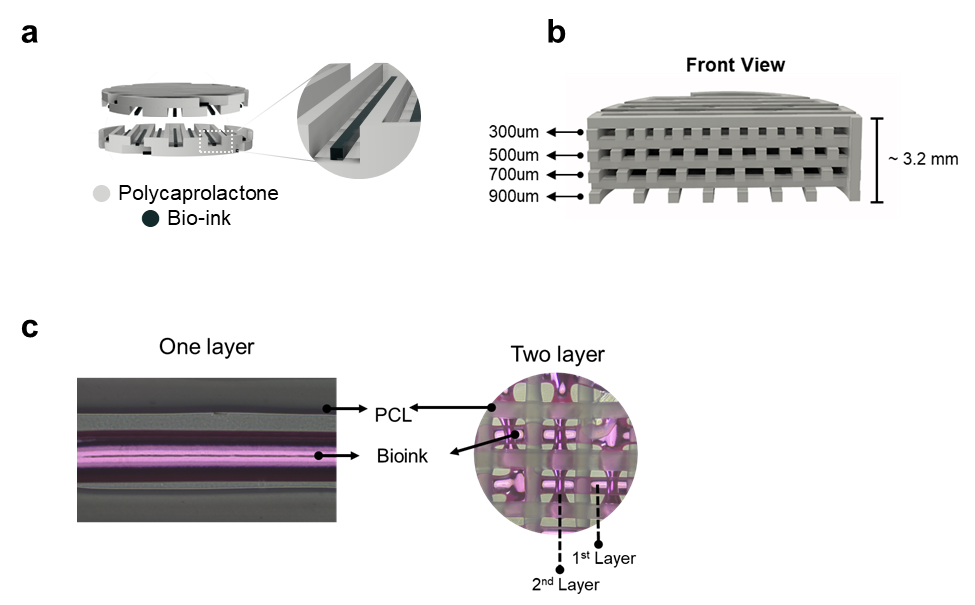


**Figure S11. Schematic and optical images of the 3D-printed osteochondral construct design.** (a) Exploded view and cross-sectional schematic showing distinct material phases. (b) Front view of the PCL frame illustrating the gradual increase of pore size. (c) Optical image of the printed PCL template with hydrogels.


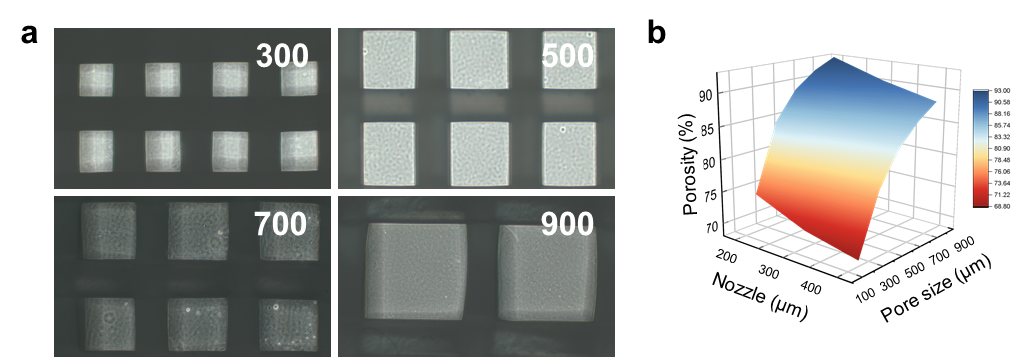


**Figure S12. Effect of designed pore size and nozzle diameter on scaffold porosity.** (a) Optical images of 3D-printed PCL scaffolds with different designed pore sizes (300, 500, 700, and 900 µm). (b) 3D surface plot illustrates the relationship between nozzle diameter, designed pore size, and measured porosity.


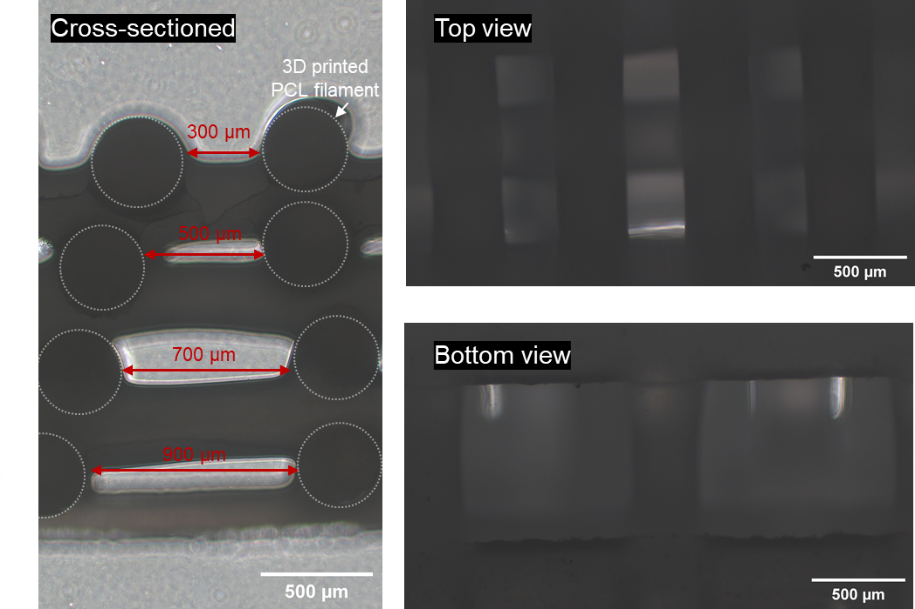


**Figure S13. Optical microscopy images of the 3D-printed osteochondral scaffold showing the preset pore architecture with gradually varied pore sizes (300–900 µm).**


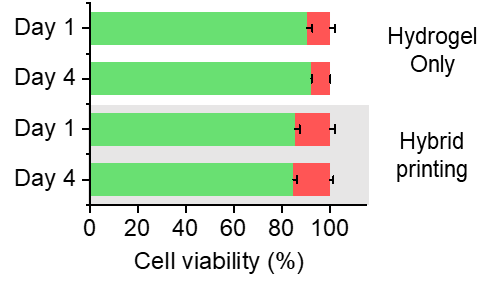


**Figure S14. Viability of hMSC spheroids in G5A1 hydrogel only and G5A1 + 300 μm hybrid printing**


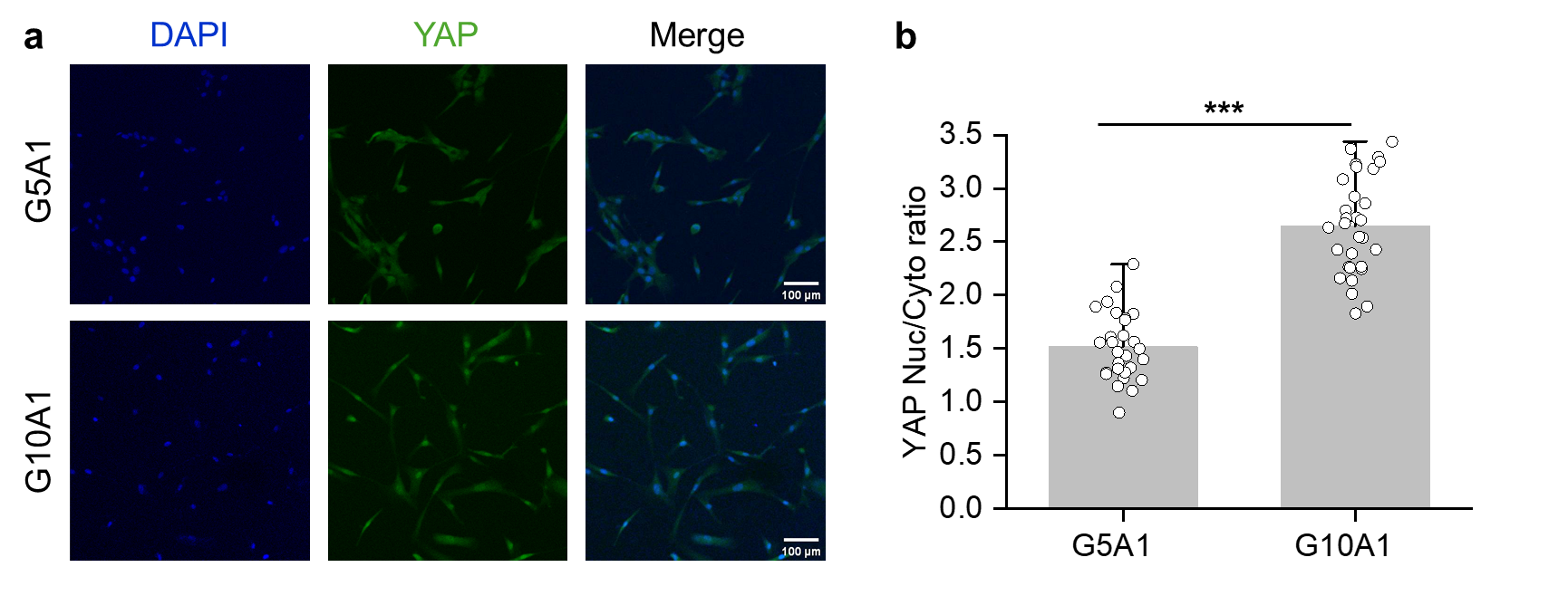


**Figure S15. Stiffness-dependent YAP localization in hMSCs.**

(a) Representative immunofluorescence images of hMSCs cultured on soft (G5A1) and stiff (G10A1) hydrogels, showing nuclei stained with DAPI (blue) and YAP (green). (b) Quantitative analysis of the nuclear-to-cytoplasmic YAP ratio confirms increased YAP nuclear translocation on G10A1 compared to G5A1, indicating stiffness-dependent mechanotransduction. Scale bars: 100 µm.


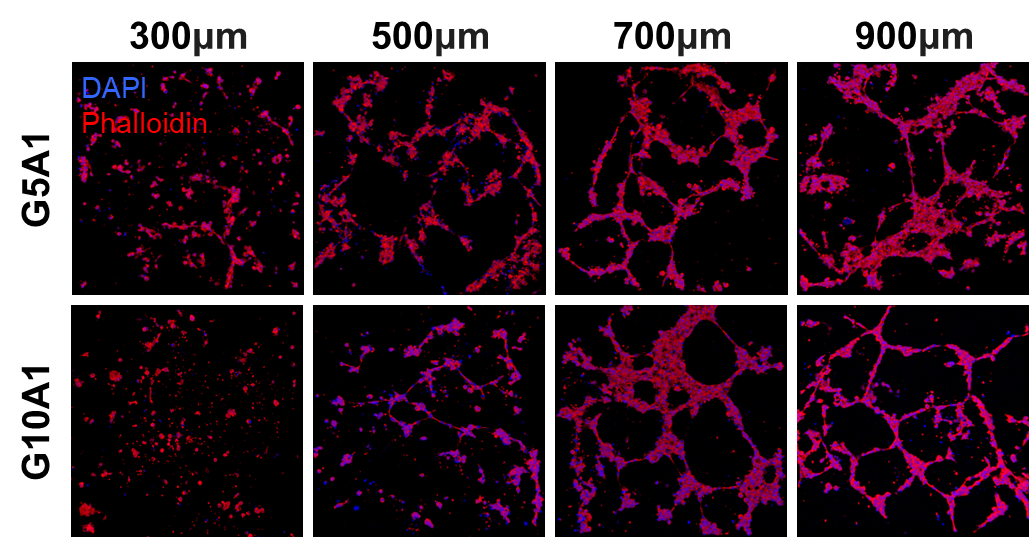


**Figure S16. Representative raw fluorescence images of tube formation assay.** Endothelial networks were visualized by staining with phalloidin (F-actin, magenta) and DAPI (nuclei, blue).


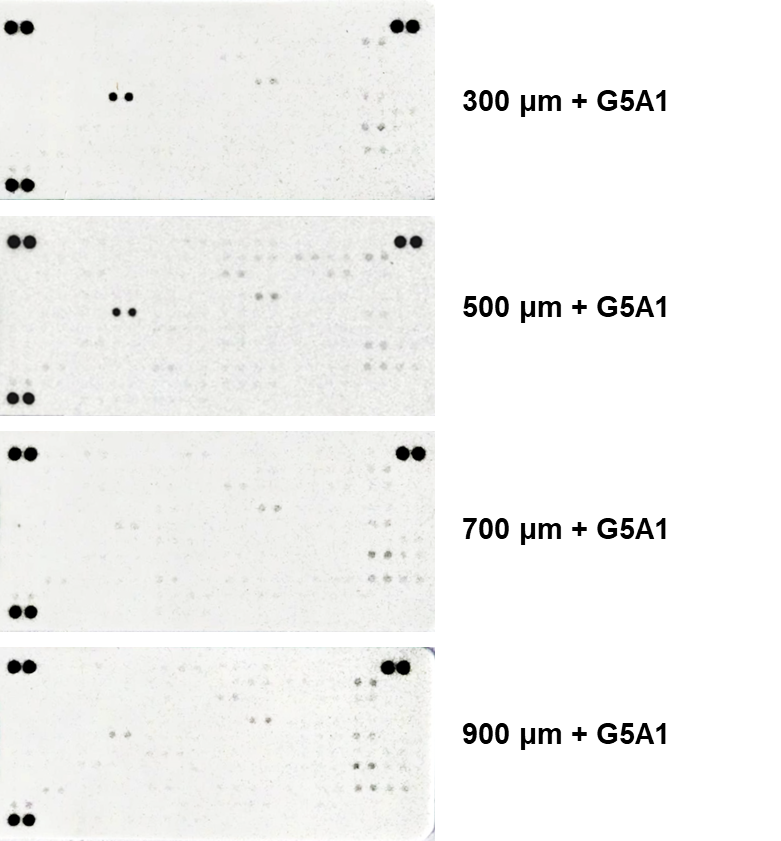


**Figure S17. The raw image of cytokine assay showing the array membrane with captured chemiluminescent signals for multiple secreted under different stiffness and porosity conditions.**

**
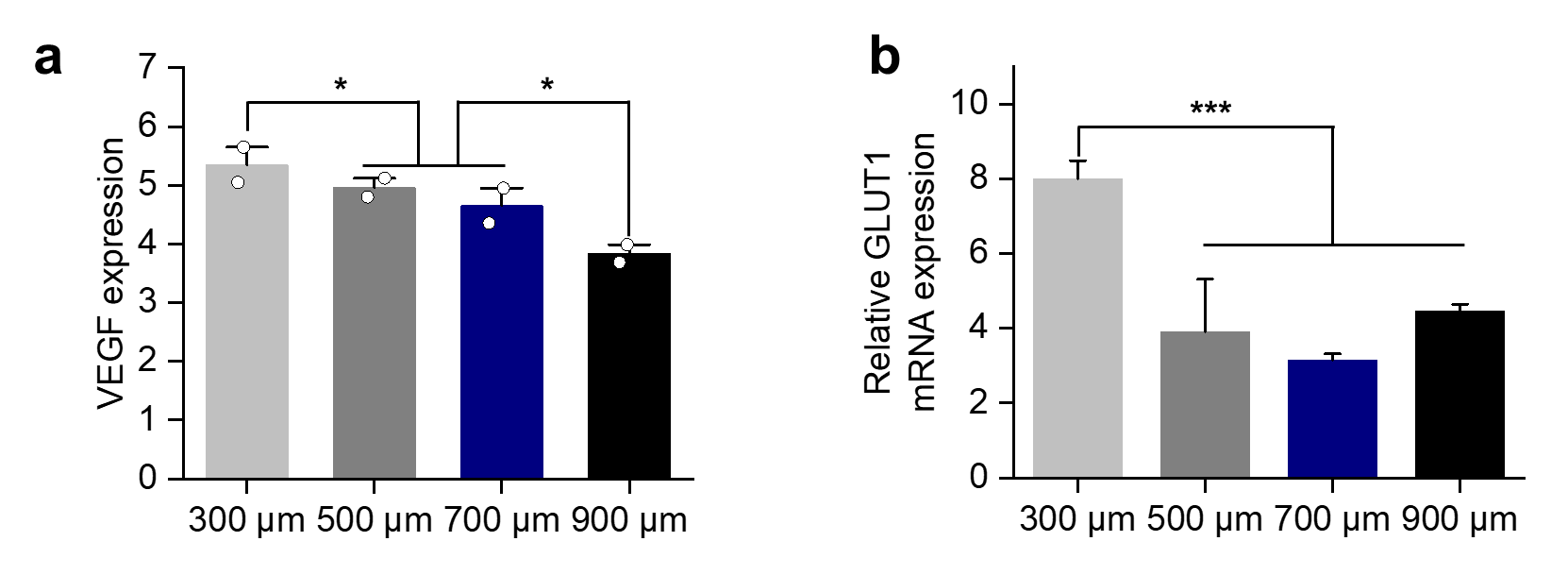
**

**Figure S18. Porosity-dependent hypoxic and metabolic signaling.**

(a) VEGF secretion, measured by cytokine analysis, in constructs with low- and high-porosity regions. (b) GLUT1 gene expression, quantified by qPCR, in low- and high-porosity regions.


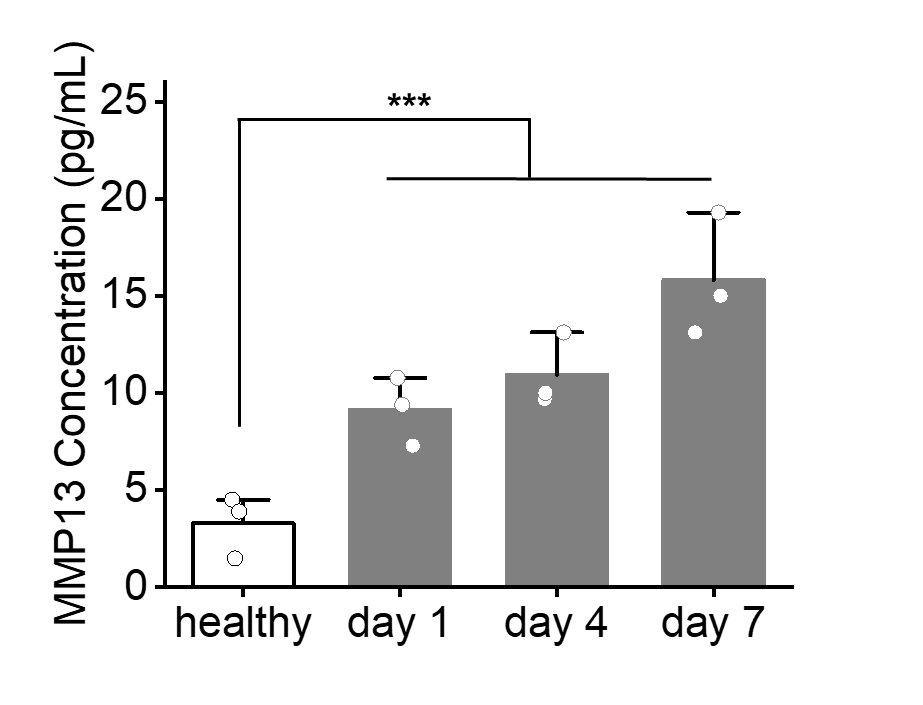


**Figure S19. Quantification of MMP-13 secretion of the bioprinted osteochondral constructs following pro-inflammatory cytokine stimulation.**


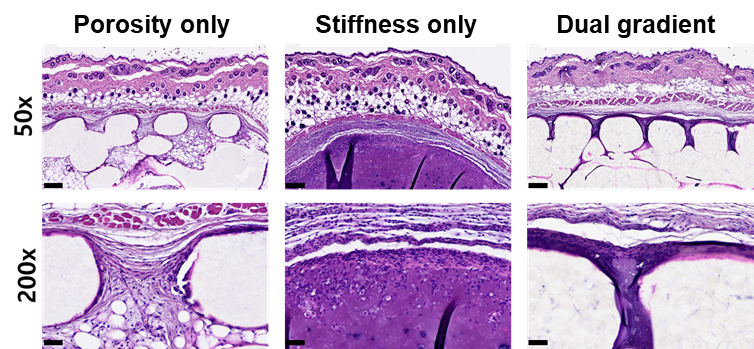


**Figure S20. Representative H&E-stained histological images of subcutaneously implanted scaffolds.** Representative H&E-stained histological images of subcutaneously implanted scaffolds. Scale bar: 200 µm (50x), 50 µm (200x).


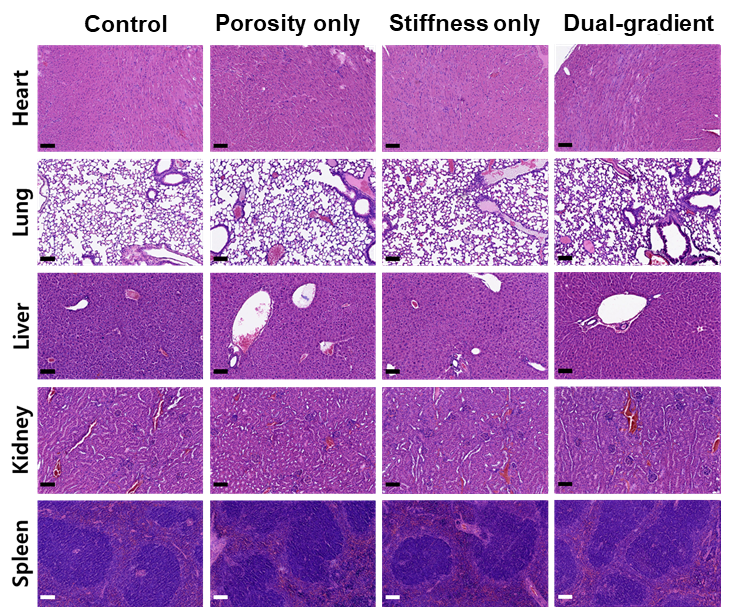


**Figure S21. Representative H&E-stained histological images of in vivo biocompatibility test.** 3 weeks after implantation. Scale bar: 100 µm.
