# Supplementary material for: Developmentally Inspired, Mechanical–Metabolic Dual Gradient Osteochondral Constructs Bridging Regeneration and Therapeutic Screening
Source: Adv Sci (Weinh). 2026 Mar 3;13(24):e16602. doi: 10.1002/advs.202516602 (PMC13116013; doi:10.1002/advs.202516602)
Supplement: Supplementary file 2 — Supporting File 2: advs74451‐sup‐0002‐TableS1.docx. [file ADVS-13-e16602-s001.docx]

**Table S1. Human primer sequences for qRT-PCR assay**

| **Gene** | | **Forward Primer** | **Reverse Primer** |
| --- | --- | --- | --- |
| **Stemness** | *CD90* | *GAAGGTCCTCTACTTATCCGCC* | *TGATGCCCTCACACTTGACCA* |
|  | *CD146* | *ATCGCTGCTGAGTGAACCACAG* | *CTACTCTCTGCCTCACAGGTCA* |
|  | *CD106* | *GATTCTGTGCCCACAGTAAGGC* | *TGGTCACAGAGCCACCTTCTTG* |
|  | *ST3GAL2* | *TCCGACTGGTTTGACAGCCACT* | *CTTCTCCAGCACCTCATTGGTG* |
| **Housekeeping genes** | *β-actin* | *TGCCCATCTACGAGGGGTATG* | *TCCTTAATGTCACGCACGATTTC* |
|  | *GAPDH* | *CCTCCTGCACCACCAACTGCTT* | *GAGGGGCCATCCACAGTCTTCT* |
|  | *18s rRNA* | *GGCCCTGTAATTGGAATGAGTC* | *CCAAGATCCAACTACGAGCTT* |
